# AgileMultiIdeogram: Rapid Identification and Visualization of Autozygous Regions Using Illumina Short-Read Sequencing Data

**DOI:** 10.3390/biology14060666

**Published:** 2025-06-09

**Authors:** Christopher M. Watson, Carolina Lascelles, Morag Raynor, Marilena Elpidorou, Ummey Hany, Laura Crinnion, Colin A. Johnson, Eamonn Sheridan, Alexander F. Markham, James A. Poulter, David T. Bonthron, Ian M. Carr

**Affiliations:** 1 North East and Yorkshire Genomic Laboratory Hub, St James’s University Hospital, Leeds LS9 7TF, UK; 2 School of Medicine, University of Leeds, St James’s University Hospital, West Yorkshire, Leeds LS9 7TF, UK

**Keywords:** rare recessive disease, next-generation sequencing, autozygosity mapping

## Abstract

Rare autosomal recessive diseases are a major cause of mortality and morbidity. They occur more frequently in individuals with consanguineous parents, in which case the pathogenic variants are often located within regions of genetic identity by descent. A well-established and effective way of identifying these “autozygous” genomic regions has been to search for runs of homozygous genotypes in microarray SNP data. However, with the widespread use of whole-genome and exome sequencing in both diagnostic and research settings, it has become desirable to be able to both map autozygous regions and identify the deleterious variants using a single dataset. We have developed AgileMultiIdeogram, an application that can identify and visualize autozygous regions in inbred individuals using exome data as well as microarray SNP genotype data. This application has been successfully used in both research and diagnostic settings to map pathogenic mutations.

## Simple Summary

The incidence of rare recessive disease is significantly increased in children whose parents are related, as both may carry the same deleterious variant inherited from a shared relative. As well as inheriting the same disease mutation, they also inherit the flanking DNA, which can be detected as extended regions of homozygous SNPs. Initially, these regions were detected using microsatellite markers, which were then replaced by microarray genotype data. With the advent of next-generation sequencing, many mutations could be found without identifying these homozygous regions; however, this failed to find the disease mutation in many patients. Consequently, there has been renewed interest in mapping these homozygous regions to help detect a patient’s deleterious mutation. Therefore, we have created AgileMultiIdeogram to identify these regions using both next-generation sequence data as well as microarray genotype data.

## 1. Introduction

Conventionally, a rare inherited disease is one affecting fewer than 5 in 10,000 individuals; however, the very large number of different conditions means that, collectively, rare diseases affect up to 8% of people [1]. As of 24 March 2025, OMIM [2] lists 6531 phenotypes with a known molecular cause mapping to an autosomal chromosome. Of these, ~55% are recessively inherited conditions, which thus constitute a major cause of mortality and morbidity.

Since first being proposed [3], autozygosity mapping has been used extensively, both in diagnosis and research, to identify the possible locations of recessive pathogenic mutations in inbred affected individuals. Such individuals generally inherit the same deleterious variant, identical by descent, along with flanking DNA, from both of their consanguineous parents. The resulting extended stretches of homozygous (autozygous) DNA may be many megabases long, and statistically, causative mutations are more likely to be located within large autozygous regions [4]. Since a child born to first-cousin parents is expected to be autozygous over 1/16 of their genome, examining a single such affected individual can exclude candidate causal variants in approximately 94% of genes. If a second affected individual within the family is included, it may be possible to exclude the vast majority of candidate variants, leading rapidly to the pinpointing of a disease-causing variant.

Initially, the advent of whole-exome sequencing (WES) and whole-genome sequencing (WGS) de-emphasized the value of mapping autozygous regions; pathogenic mutations could often be identified simply by scanning a patient’s variant data for putatively deleterious homozygous variants within the coding sequences of genes believed to be important in the pathways disrupted in the patient. However, while this approach has been effective in many families, a significant number of cases remain unresolved and would potentially benefit from information regarding the likely genomic position of the causative variant. Consequently, such cases are being revisited with their autozygous regions mapped and used to filter their variants. Historically, autozygous regions were defined using microsatellite markers and later by microarray SNP genotyping. However, it is now advantageous to be able to identify autozygous regions directly from the patient’s WGS or WES data.

As a consequence, a number of applications, such as AgileVariantMapper [5], H^3^M^2^ [6], PLINK [7], AutoMAP [8], AUDACITY [9], SavvyHomozygosity and SavvyVcfHomozygosity from the SavvySuite [10] and AutozygosityMapper [11], were developed to aid the identification of autozygous regions using WES and WGS variant data. The process by which some of them were validated is not apparent. As of 1 June 2025, SavvyHomozygosity and SavvyVcfHomozygosity are cited as a GitHub repository primarily concerned with copy number analysis. PLINK and AutozygosityMapper are established applications to which the ability to process WES and WGS has been added but not clearly described in subsequent publications. The manuscript describing AgileVariantMapper lacks a rigorous comparison and simply compares the visualization of regions identified using WES and microarray SNP genotype data. H^3^M^2^ was developed using three reference cell lines, whose data were subsequently used as the starting point for the production of a range of synthetic data used in the validation step. Meanwhile, AUDACITY was validated using 200 samples from the 1000 Genome Project [12], whose autozygous regions were identified using PLINK, BCFtools [13] and VCFtools [14]. AutoMap was validated using 52 samples for which WES and microarray SNP genotype data were available. This cohort was then split into a training set of 26 samples and a test set consisting of the remaining 26 samples.

Microarray SNP genotype data are generally considered the gold standard for the detection of autozygous regions, as they consist of a predetermined set of evenly distributed, pre-validated variant positions whose genotypes are determined with a high degree of certainty. Compared to microarray SNP genotype data, detecting autozygous regions in WGS or WES is a more complex task because sequence data are more error-prone, and in the case of WES, unevenly distributed across the genome. Consequently, it is the preferred method for the detection of autozygous regions when processed by a suitable application such as PLINK, which has approximately 35,000 citations covering the analysis of microarray genotype data. While synthetic WES and WGS data were used to validate H^3^M^2^, it is debatable whether the synthetic data fully reflect the random noise present in WGS and WES datasets, and so it may not be a suitable resource for this validation. Similarly, AUDACITY was validated against a set of regions identified using PLINK, VCFtools and BCFtools with the WES data; however, none of these tools is widely used to determine autozygosity and so they may not have captured all the regions in each sample. Consequently, of the tools listed, only AutoMap can be considered to be fully validated; unfortunately, for ethical reasons, the authors are unable to share their data, and so it cannot be used to validate other applications.

Of the listed applications, only AutozygosityMapper and AutoMap generate images; however, their format is not always suitable for the display of regions in a publication and does not allow the use of a range of data types such as microarray SNP genotype and WES and/or WGS variant data. Consequently, we have developed a new adaptive algorithm that allows the automated detection of autozygous regions within WES and WGS, as well as genotype data from a range of SNP microarrays. When processing WES and WGS data, this algorithm re-genotypes variants and identifies and filters erroneous heterozygous variants located within runs of homozygous variants. This algorithm was then implemented in the desktop application AgileMultiIdeogram, which can visualize the autozygous regions in a cohort of samples whose data consists of a mix of microarray SNP genotyping, WES and/or WGS variant datasets.

Due to the very large number of possible input parameter combinations, a genetic algorithm was used to determine the near-optimal parameters required to detect autozygous regions from sequencing data. Genetic algorithms represent a methodology for determining the near-optimal values for parameters used in an analysis. An iterative process inspired by evolution is used. In evolution, individuals mate and only the fittest offspring survive to reproduce in the next generation, after which the process repeats. In theory, this process increases the fitness of the population until an optimum set of characteristics is held by the offspring. Similarly, genetic algorithms perform data analysis many times using randomly selected parameters, with the results of each analysis being quantified. The best-performing parameter sets are selected and used to create a new generation of parameter sets, which are in turn used to reprocess the data, with the best-performing parameters again passed on to the next generation. At each step, “mutations”—random changes—are introduced along with new datasets so that the final result is not solely determined by the values in the initial generation. After multiple iterations, a stable set of near-optimal parameter values should occur.

## 2. Materials and Methods

### 2.1. Patient Data

Twenty-two individuals with consanguineous parents and one outbred person were analyzed by exome sequencing and SNP array genotyping as described below. The consanguineous parents of these individuals were first cousins, suggesting that the individuals are autozygous for approximately 1/16 of their genome. However, the extended pedigrees were generally characterized by multiple additional consanguineous unions, making the true level of autozygosity difficult to predict, but probably higher than 1/16. Of these individuals, 18 (including the outbred person) were used as the training dataset, which was used by the genetic algorithm to identify the optimal parameter values, while the remaining 6 were used as a test dataset, comparing the accuracy of the outcome to those produced by H^3^M^2^, PLINK, AutoMAP, AUDACITY, SavvyHomozygosity and SavvyVcfHomozygosity from the SavvySuite and AutozygosityMapper in identifying variants in autozygous regions. Each individual’s dataset consisted of paired-end short-read data of between 40 and 50 million read pairs per subject.

### 2.2. Exome Sequencing and Variant Detection

Following shearing to approximately 250 bp, 3 µg of genomic DNA was used to create Illumina-compatible libraries that were enriched for coding sequences using Agilent’s v5.0 SureSelect exome reagent. Five individuals were sequenced per HiSeq 2500 high-output lane to generate 100 bp paired-end read data, which were then aligned to the human genome (hg19) using BWA after quality trimming using Cutadapt [15]. Sequence variants were identified using GATK [16] and exported to VCF files. Where possible, each variant was linked to an RS ID using BCFtools and the 1000 Genomes variant dataset [12,17] as the reference variant dataset.

### 2.3. Identification of Autozygous Regions with Exome Data Using PLINK, H^3^M^2^, SavvyHomozygosity, SavvyVCFHomozygosity, AUDACITY, AutozygosityMapper and AutoMAP

Each subject’s autozygous regions were predicted from exome variant data using each of the above-named methods, as follows:

PLINK: the required MAP and genotype files were generated from the VCF files and processed using PLINK 1.07 with the suggested parameters (--homozyg --homozyg-snp 100 --homozyg-window-het 1 --homozyg-window-snp 50 --homozyg-window-threshold 0.10).

H^3^M^2^: Rather than use the variant data determined by GATK, the analysis was performed on the aligned data in the BAM files. Initially, the H3M2BamParsing.sh script was used to parse each BAM file with reference to a list of variants (obtained from the H^3^M^2^ website) to produce a list of predefined variant genotypes. These data were then processed using the H3M2Analyze.sh script with the suggested parameters (dnorm = 1,500,000, p1 = 0.1, p2 = 0.2 and F = 5) to identify the autozygous regions.

AutoMAP: the AutoMAP_v1.2.sh script was used to process each VCF file in turn using the pre-set settings.

SavvyHomozygosity and SavvyVCFHomozygosity: These applications require a reference set of known variants, which for this analysis were derived from the first hundred individuals in the 1000 Genomes Project variant dataset. Deletions and insertions longer than 1 bp were removed, as were variants with fewer than 2 non-homozygous reference genotypes. These data were then formatted using the SavvySuite’s PrepareLinkageData application to produce a binary file of the variants. As with H^3^M^2^, SavvyHomozygosity processes the sample’s aligned sequence data, while SavvyVCFHomozygosity filters predetermined variants in the sample’s VCF file against the list of known variants. The filtered variants are then used to determine the location of homozygous regions. Both SavvyHomozygosity and SavvyVCFHomozygosity were used with their pre-set parameters.

AUDACITY: The analysis consists of two Perl scripts: The first (AUDACITYPrepare.pl) filters the variants to remove indels and multiallelic variants and determines the number of each genotype present in the dataset (homozygous reference, heterozygous and homozygous non-reference). The retained variants are then stored in an indexed bgzip-compressed VCF file that is processed by the second script. This script (AUDACITYAnalyze.pl) processes the variant dataset with reference to a list of known variants to determine the location of any homozygous regions.

AutozygosityMapper: The VCF files were sequentially uploaded to the AutozygosityMapper data entry web page and then analyzed individually using the default settings. The regions were copied from the results page and ordered by chromosome and position before saving them as tab-delimited text files.

Since these applications, as well as AgileMultiIdeogram, are intended to identify extended regions of autozygosity due to recent inbreeding, regions shorter than 1.5 Mb were discounted, while closely adjacent (<100 kb) regions were amalgamated into a single region.

### 2.4. Microarray SNP Genotype Data Production

Autozygous regions were identified in each patient using the microarray genotype data as follows: Affymetrix SNP 6.0 microarray data (Aros Applied Biotechnology A/S, Aarhus, Denmark) were generated for each patient with autozygous regions identified using PLINK 1.07 (--homozyg--homozyg-window-snp 250--homozyg-window-het 2--homozyg-snp 300 --homozyg-kb 1000). The data were then visualized in AutoSNPa [18] to identify regions missed due to low-quality SNP genotyping calls, and finally the regions were manually edited to remove regions shorter than 1.5 Mb, while closely spaced (>100 kb) regions were combined into a single region.

## 3. Results

### 3.1. The Algorithm to Detect Autozygous Regions

**Figure 1:**
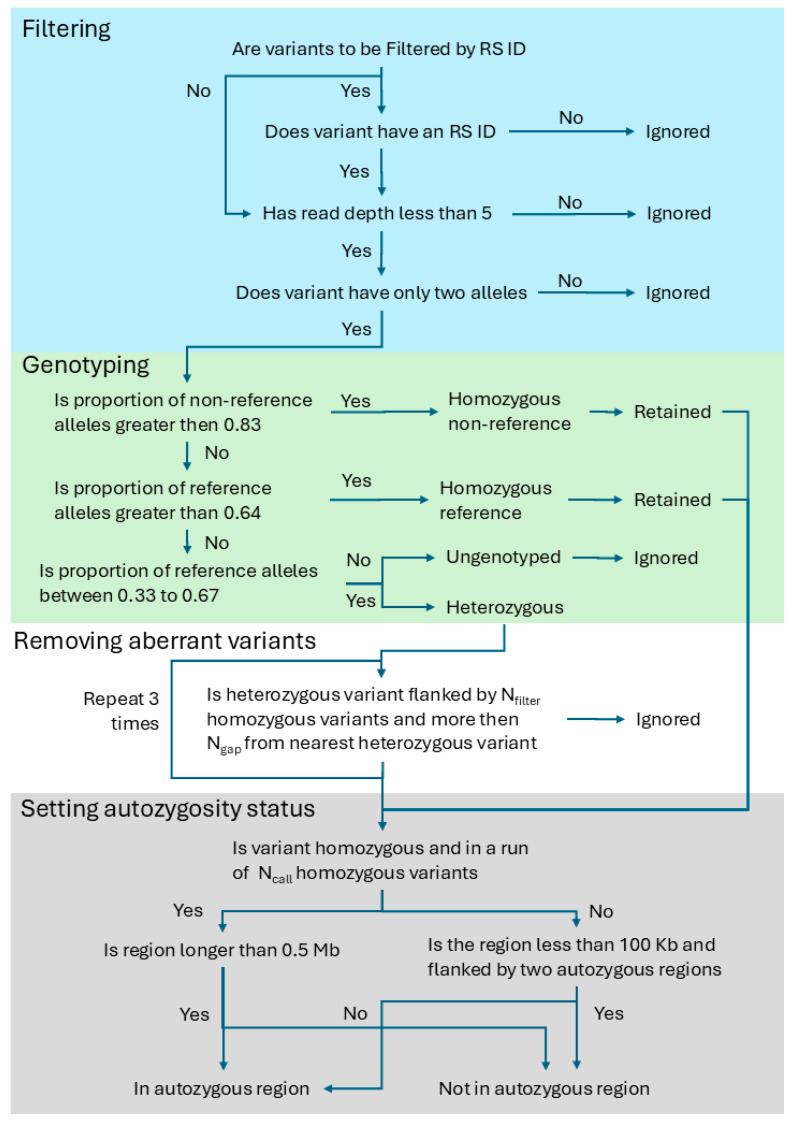
Flowchart describing the process by which the autozygosity of a variant is determined. The process is split into four parts: variant filtering (blue section), variant genotyping (green section), removal of aberrant heterozygous variants (white section) and determining autozygosity (grey section).

To compensate for the effects of genotyping errors, the AgileMultiIdeogram algorithm that detects autozygous regions applies empirically determined smoothing criteria to the exome variant data. Also, to make the algorithm responsive to the different variant densities that characterize different individual next-generation sequencing (NGS) datasets, the number of variants required in homozygous runs is scaled using the total number of variants on Chromosome 1, relative to an arbitrary value (70,700: the approximate number of Chromosome 1 SNPs in the Affymetrix SNP6 dataset). Unlike microarray genotype data, the genotypes of individual SNVs in WGS or WES datasets are of uncertain quality (for example, due to variations in read depth and the quality of each read); consequently, each variant’s genotype is reassessed, and putatively erroneous heterozygous variants are discounted before the data are screened for autozygous regions using cut-off values (see Table 1) scaled to the number of variants in the dataset, as shown in Figure 1 and described in the supplementary data document.

The process consists of four sections: basic filtering, genotyping, removal of aberrant heterozygous variants and finally setting the autozygosity of a region. Initially, variants undergo a basic filtering process: if a variant is required to have an RS id or is not a single base variant, it is ignored. Then, variants that have more than two alleles or a read depth below 5 (N_R_) are ignored. The remaining variants are then genotyped and the proportion of reads suggesting each allele is determined. If the proportion of reads linked to the non-reference allele is 0.83 (1 − N_BB_) or greater, the variant is homozygous. The variant is considered heterozygous if the reference allele is present in 0.33 to 0.67 (0.5 ± N_het_) of the reads. Finally, if the proportion of reads linked to the reference allele is 0.64 or more, the variant is homozygous. Ungenotyped variants are discounted. The optimized values of each parameter used to analyze user data are shown in the final column of Table 1.

### 3.2. Parameter Optimization by Simulated Evolution

Initially, 100 sets of parameters were generated, with values constrained as outlined in Table 1. Each parameter set was then used to process the exome genotype data for the 18 subjects in the training data, for whom microarray SNP genotype data were also available. The resulting autozygous regions were then compared to the manually curated list of autozygous regions generated by PLINK from the Affymetrix SNP6 genotype data, as described in the Materials and Methods section. For each Mb over which the two lists of autozygous regions differed, the analysis scored +1, with the scores for each subject combined to give a final score for each parameter set. Any analysis scoring less than or equal to the tenth-best analysis was retained for the next generation: for this, the retained parameter sets were randomly “mated” to the other retained parameter datasets, such that each parameter in the new set was randomly selected from one of the parents; to increase variation, there was also a 1 in 5 chance that each parameter would be overwritten by a randomly selected value. When necessary, randomly generated parameter sets were added to each generation, such that all generations had at least 100 parameter sets. This genetic selection algorithm was run for a period of 48 h (approximately 40 to 45 generations). Since any single analysis will still be influenced by its starting values, this entire process was run 100 times, after which no noticeable improvements were obtained, with the optimum values identified as the best set from all the analyses shown in the final column of Table 1. It is expected that these values will be used for all subsequent analysis.

### 3.3. Comparison of AgileMultiIdeogram, H^3^M^2^, AutoMAP, PLINK, AutozygosityMapper, SavvyHomozygosity and SavvyVCFHomozygosity

**Figure 2:**
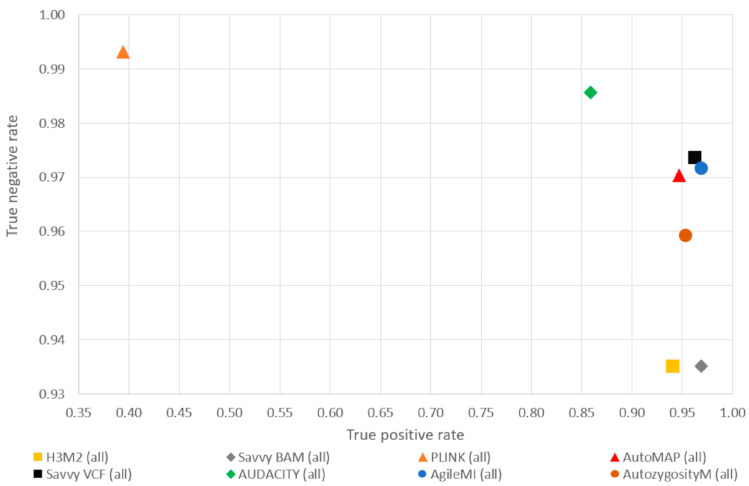
Graph demonstrating the true positive and true negative rates of variant classification using autozygous regions identified by AgileMultiIdeogram (blue symbols), AutoMAP (red symbols), H^3^M^2^ (yellow symbols), SavvyHomozygous (grey symbols), SavvyVCFHomozygous (black symbols), AutozygosityMapper (brown symbols) and PLINK (orange symbols) using exome data. Each symbol represents the aggregated score of the 6 inbred individuals.

A comparison of the regions identified by PLINK (using microarray genotype data) to those identified by each of the other programs was performed using the six samples in the test dataset to determine the optimal parameters. Variants in regions identified by PLINK using microarray data were classed as positive, while those outside the regions were classed as negative. This was repeated using the regions defined by each of the applications; where a variant’s status was in agreement with PLINK, it was classified as a true positive (both methods suggested it was autozygous) or true negative (both methods suggested it was not autozygous). When the two analyses disagreed, the variant was classified as either a false negative (the variant was determined to be autozygous only by PLINK using microarray genotype data) or false positive (the variant was assessed as not autozygous only by PLINK using microarray genotype data). This allowed the counts of true and false positives as well as true and false negatives to be determined for each method (Table 2 and Supplementary Table S1), which were then used to calculate the true positive rate (sensitivity) and true negative rate (specificity) for each individual and for the dataset as a whole (Figure 2 and Supplementary Figure S1). The results of the comparison show that AUDACITY, H^3^M^2^, SavvyHomozygosity and PLINK performed the worst when both sensitivity and specificity are considered. When considering the true negative rate, there was very little difference between AutoMAP (0.9704), AgileMultiIdeogram (0.9716) and SavvyVCFHomozygosity (0.9736), while for the true positive rate, AgileMultiIdeogram (0.9692) and SavvyVCFHomozygosity (0.9632) performed noticeably better than AutoMAP (0.9471).

### 3.4. Implementation

**Figure 3:**
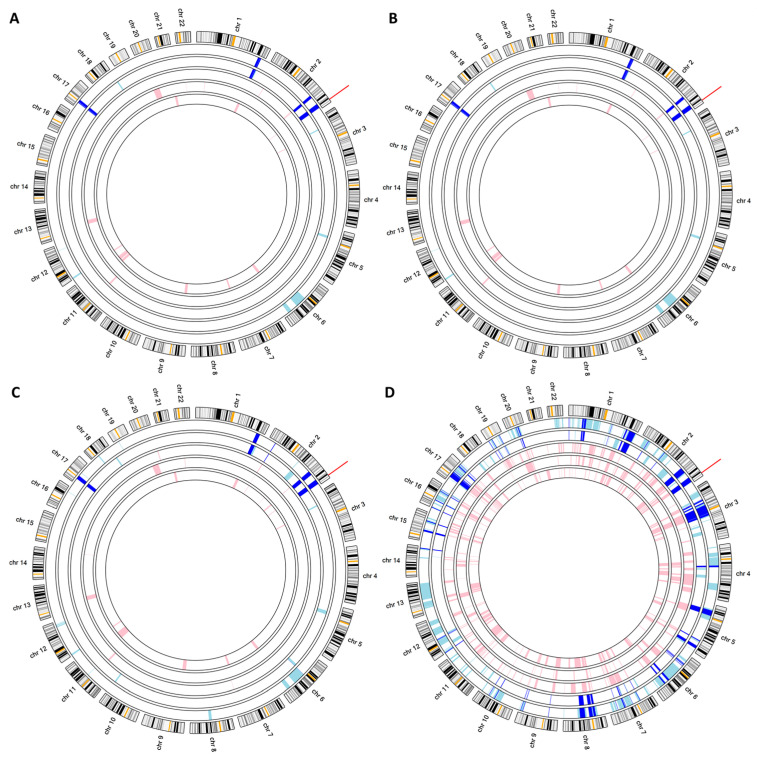
Aligned autozygous regions in a family of four siblings and their parents, in which two siblings are affected by the lethal multiple pterygium syndrome (MIM 253290). (**A**,**B**) were generated using whole exome data, while (**C**,**D**) were derived from whole genome data. (**A**,**C**) only used variants with an RS ID, while (**B**,**D**) used all variants. Autozygous regions in affected and unaffected individuals are shown as pale blue and pink bands, respectively. Dark blue bands indicate autozygous regions common to all affected individuals. The red line marks the position of the *CHRNG* gene. The individuals from the outside to the center are affected male sibling, affected female sibling, unaffected female parent, unaffected male parent and unaffected female sibling.

AgileMultiIdeogram is a Windows desktop application written in C# that, while aimed at the Windows desktop, can also run on Linux and macOS computers with the aid of Wine: https://www.winehq.org/ (accessed on 1 June 2025). AgileMultiIdeogram can detect and visualize autozygous regions within various data types, such as microarray SNP genotype data and Illumina short-read sequencing variant data formatted as either VCF or gVCF files, as well as predetermined regions imported as tab-delimited text files. Any identified regions can be exported as plain text or as a range of publication-quality images [19,20,21,22,23,24,25,26]; in the latter case, either circular or linear ideograms can be chosen (Figure 3 and Supplementary Figure S2). For flexible integration of different information when a family may have been analyzed “piecemeal”, AgileMultiIdeogram also has the ability to process microarray SNP genotype type data as well as display regions identified by other applications and imported as tab-delimited text files. Since it is not possible to redetermine the genotypes of variants in microarray SNP genotype data, this type of data enters the analysis at step 7, where aberrant heterozygous variants in long runs of homozygous variants are detected and removed from the analysis. A preliminary investigation to determine the optimal parameters for use with microarray SNP genotype data suggested that the analysis was not especially sensitive to their values; consequently, the same values were used for microarray SNP genotype data as were used with exome variant data. This is demonstrated in Supplementary Figure S3, which shows the regions identified using WES variant and microarray SNP genotype data for each of the samples used in the training and testing set.

As part of the parameter optimization process, the underlying algorithm used to identify the autozygous regions was implemented as a C++ console application that runs on Linux and Windows computers. This allows variant data in VCF files to be processed as part of an automated pipeline. The source code for this implementation is available here: https://github.com/msjimc/AgileROH (accessed on 30 May 2025).

## 4. Discussion

Short-read WGS and WES have rapidly become an almost universal entry point for genetic investigations in both research and diagnostic settings. These agnostic approaches often allow direct identification of pathogenic genetic lesions, without recourse to other approaches such as genetic mapping or biochemical assays. Nonetheless, for a substantial proportion of patients, an obviously pathogenic lesion does not stand out from the set of identified variants. In such cases, particularly in diagnostic settings where costs are constrained, further analysis may be discontinued in favor of prioritizing the testing of other patients with more easily identifiable mutations. It remains the case, though, that many such cases could be resolved by minimal additional analysis tailored to the individual context. For this purpose, the revisiting of older approaches such as genetic mapping of disease loci can be valuable, particularly for rare recessive disorders. It is advantageous to be able to use pre-existing sequencing data for such cases, rather than perform additional microarray genotyping. However, due to the lower genotyping accuracy of these short-read datasets (and, in the case of WES, the uneven genomic distribution), identifying autozygous genomic regions is more error-prone than when using microarray genotype data [5] (Carr et al., 2013). Because these sources of error are experimental rather than statistical, there is insufficient evidence on which to base a mathematical solution to this problem. We have therefore investigated the utility of a genetic algorithm approach to empirically select the optimal parameter values for detecting autozygous regions. By an iterative process of comparing the results from different parameter sets and combining the best-performing sets, it has proved possible to identify a set of near-optimal analysis parameters for the discovery of autozygous regions using only exome variant data.

While it would have been preferable to use a larger training and testing dataset, patient data with both microarray and WES/WGS data are not readily available. For instance, due to ethical considerations, the dataset used to develop AutoMap is not available. Similarly, raw data in large biobanks, such as UK Biobank [27], are available for download and can only be processed within the UK Biobank data analysis portal. The creation and use of synthetic data is commonly used in the development of bioinformatics programs such as H^3^M^2^; however, this is not appropriate when dealing with WES and WGS variant datasets. Due to sequencing errors and sequence variation between the patient and reference genome sequences, the location and quantity of aberrant variant calls cannot easily be modeled. This is compounded by reads from highly homologous duplicated sequences being misaligned and so creating hotspots of incorrect variant calls. Similarly, hotspots may occur at locations with unusual GC content, which present as regions of low sequencing depth. Consequently, the creation of synthetic data based on the overall genotyping error rate of NGS techniques is not suited to the analysis of WES/WGS variant data, as the resultant data are unlikely to represent the true granularity of aberrant genotyping across the genome. The developers of AUDACITY chose to identify the autozygous regions in patient data by analyzing them with a combination of three other applications (PLINK, VCFtools and BCFtools); however, this methodology will always miss regions that are inherently resistant to NGS-based analysis and so will not truly reflect the accuracy of the method under test.

Microarray SNP genotype data consist of the genotype data of up to 1 million preselected variant positions and has been the foundation of both genome-wide association studies and disease gene mapping studies since its introduction. Unlike NGS variant data, microarrays consist of highly curated positions chosen for their distribution, heterozygosity and high level of reducibility. Consequently, detecting autozygous regions is a trivial task possible by simply scanning a region by eye. However, due to the number of data points in a typical chip design, a number of applications were developed to automate the process, such as PLINK. Consequently, when developing and validating AgileMultiIdeoigram, it was decided to determine the autozygous regions in the test subjects using Affymetrix microarray SNP6 genotype data processed using PLINK.

When the performance of our optimized algorithm was compared to that of other applications (using samples not used in the optimization process), AgileMultiIdeogram achieved the best true positive ratio and a true negative rate just 0.0216 below the best true negative rate, which was achieved by PLINK. However, PLINK’s true positive rate was 0.3641, compared to 0.9692 for AgileMultiIdeogram. When both the true positive and true negative rates are considered, PLINK and AUDACITY achieve high true negative rates but at the expense of a poor true positive rate; conversely, H^3^M^2^ and SavvyHomozygosity have good true positive rates at the expense of poorer true negative rates. AgileMultiIdeogram, AutoMAP and SavvyVCFHomozygosity formed a cluster with similar high scores for both true positive and true negative rates, suggesting that AgileMultiIdeogram is able to robustly detect autozygous regions using exome variant data.

Unlike the other applications, AgileMultiIdeogram is able to detect autozygous regions using data from WGS, WES and microarray SNP genotype experiments, as well as import regions identified by other methods when generating publication-quality figures. Since WGS and WES data only report positions that differ from a reference sequence, their data tend to only contain heterozygous or homozygous non-reference genotypes. However, when the variants are recalled by AgileMultiIdeogram, some variants (particularly heterozygous variants) are determined to be, in fact, homozygous reference. For WES data, this is generally not an issue, but due to the lower read depths currently associated with WGS data, a sizable proportion of a WGS variant dataset may be reset to homozygous reference, causing the AgileMultiIdeogram algorithm to fail due to an excess of recalled homozygous positions. This issue can be overcome by allowing AgileMultiIdeogram to exclude variants that lack an RS identifier in the input data since variants that lack an RS id are more likely to be artefactual than those with an RS id (Figure 3).

The memory requirements of AgileMultiIdeogram are modest; since it only retains a variant’s position and genotype data when processing each sample, the program can be run on a standard desktop computer. The time required to process each file is linearly dependent on the number of variants in the dataset. Typically, a WES VCF file is processed in several seconds, while a microarray SNP genotype file may require 10 to 20 s. Due to the large size of WGS variant data files, these may take several minutes to process.

An important practical advantage of AgileMultiIdeogram is its input data flexibility. While it performs comparably to or better than a range of other software, AgileMultiIdeogram can process a wider range of input data types (including either microarray SNP genotype data or WGS/WES variant data) as well as detect common autozygous regions within a set of affected and unaffected individuals. For users who may prefer to use regions identified by another application, it is possible to import regions as a tab-delimited text file and then prompt AgileMultiIdeogram to create the desired image. While the desktop AgileMultiIdeogram application itself may not be suitable for use in an automated pipeline, its underlying algorithm was ported to the C++ language as part of the parameter optimization process. It is possible to compile this application to run from either Windows or Linux command lines, enabling it to be incorporated into automated pipelines.

## 5. Conclusions

Careful analysis of NGS variant datasets can identify deleterious variants in a large proportion of autozygous patients afflicted by a recessive disease without the need to map their autozygous regions, especially if there is a strong candidate gene. However, for a significant number of individuals, this approach fails; as a result, there has been a renewed interest in mapping autozygous individuals who have not received a molecular diagnosis. In many situations, these patients will have been routinely sequenced, and so it is advantageous to be able to use these datasets to map their autozygous regions. To facilitate this, we have developed an algorithm that can detect autozygous regions using either NGS variant or microarray genotype data. Its performance is on par with the best autozygous mapping applications when using NGS data. To increase its utility, it has been implemented in a command line application that can be used as part of an automated analysis pipeline and a desktop application that can draw publication-quality images of the detected regions in multiple individuals.

## Figures and Tables

**Table 1:** Starting values used by the genetic algorithm to optimize the AgileMultiIdeogram algorithm. N_R_ is the minimum read depth, N_AA_ is the proportion of reads suggesting the reference allele for a homozygous reference genotype, and N_BB_ is the proportion of reads suggesting the reference allele for a homozygous non-reference genotype. N_het_ is the divergence from 0.5 allowed for a heterozygous genotype (0.5 ± N_het_). The X_filter_ constant is used to set the minimum number of flanking homozygous variants before a heterozygous variant is discounted.

Parameter	Range of Tested Values	Minimum Change in Value	Final Optimized Value for All Subsequent Use
N*_R_*	0–19	1	5
N*_AA_*	0.56–0.95	0.01	0.64
N*_BB_*	0.05–0.34	0.01	0.17
N_het_	0.05–0.34	0.01	0.17
X_filter_	75–574	1	386
X_minimum_	0.01–0.50	0.01	0.1
X_call_	300–1499	1	575

**Table 2:** The number of variants found to reside in and outside of autozygous regions identified by AgileMultiIdeogram, AutoMAP, H^3^M^2^, SavvyHomozygosity, SavvyVCFHomozygosity, AUDACITY, AutozygosityMapper and PLINK using exome variant data was compared to the same variants similarly classified with a manually curated list of autozygous regions generated using Affymetrix SNP6 genotype data to quantify the number of correctly classified variants. These values were used to calculate the true positive rate (TPR) and true negative rate (TNR) for each methodology.

Method	True Positives	False Positives	True Negatives	False Negatives	TPR	TNR
Agile- MultiIdeogram	22,821	9214	315,182	725	0.9692	0.9716
PLINK (VCF)	9279	2202	322,194	14,267	0.3941	0.9932
AutoMAP	22,301	9612	314,784	1245	0.9471	0.9704
H^3^M^2^	22,158	21,094	303,302	1388	0.9411	0.9350
Savvy- Homozygosity	22,820	21,026	303,370	726	0.9692	0.9352
Savvy- VCFHomozgosity	22,680	8572	315,824	866	0.9632	0.9736
AUDACITY	20,231	4673	319,723	3315	0.8592	0.9856
Autozygosity- Mapper	22,470	13,261	311,135	1076	0.9543	0.9591

## Data Availability

The data associated with this paper are available on request from the University of Leeds Data Repository: https://doi.org/10.5518/1197. User guides, source code, compiled programs and sample VCF files are freely available at https://github.com/msjimc/AgileMultiIdeogram (accessed on 30 May 2025) and https://github.com/msjimc/AgileROH (accessed on 30 May 2025).

## Supplementary Materials

The following supporting information can be downloaded at: https://www.mdpi.com/article/10.3390/biology14060666/s1, Figure S1: Graph demonstrating the true positive and true negative rates of variant classification using autozygous regions identified by various programs; Figure S2: An ideogram drawn with linear chromosomes on which aligned autozygous regions in a family of four siblings and their parents are shown.; Figure S3: 3 Aligned autozygous regions for the samples used in the training and testing set. The outer, blue regions are derived from WES data, while the inner pink segments were determined using microarray SNP genotype data. Table S1. Lists of variants found to reside inside and outside of autozygous regions identified by AgileMultiIdeogram, AUDACITY, AutoMAP, AutozygosityMapper, H^3^M^2^, PLINK using exome variant data were compared to the same variants similarly classified by PLINK using Affymetrix SNP6 genotype data, SavvyHomozygosity and SavvyVCFHomozygosity, to quantify the number of correctly classified variants. These values were used to calculate the true positive rate (TPR) and true negative rate (TNR) for each consanguineous individual’s data.

## Abbreviations

The following abbreviations are used in this manuscript:

WES	Whole-exome sequencing
WGS	Whole-genome sequencing
NGS	Next-generation sequencing
