# Supplementary material for: AgileMultiIdeogram: Rapid Identification and Visualization of Autozygous Regions Using Illumina Short-Read Sequencing Data
Source: Biology (Basel). 2025 Jun 9;14(6):666. doi: 10.3390/biology14060666 (PMC12189163; doi:10.3390/biology14060666)

Individual	Method	True Positives	True Negatives	False Positives	False Negatives	TPR	TNR
A	AgileROH	5,671	49,368	1,599	193	0.9671	0.9686
A	AUDACITY	4,807	50,796	171	1,057	0.8197	0.9966
A	AutoMAP	5,688	49,771	1,196	176	0.9700	0.9765
A	AutozygosityMapper	5,579	47,547	3,420	285	0.9514	0.9329
A	H3M2	5,546	48,211	2,756	318	0.9458	0.9459
A	PLINK (VCF)	2,259	50,943	24	3,605	0.3852	0.9995
A	Savvy Bam	5,579	47,547	3,420	285	0.9514	0.9329
A	Savvy VCF	5,580	50,451	516	284	0.9516	0.9899
B	AgileROH	1,802	53,697	3,435	9	0.9950	0.9399
B	AUDACITY	1,743	55,187	1,945	68	0.9625	0.9660
B	AutoMAP	1,804	53,966	3,166	7	0.9961	0.9446
B	AutozygosityMapper	1,743	55,187	1,945	68	0.9625	0.9660
B	H3M2	1,734	52,157	4,975	77	0.9575	0.9129
B	PLINK (VCF)	822	56,046	1,086	989	0.4539	0.9810
B	Savvy Bam	1,785	52,364	4,768	26	0.9856	0.9165
B	Savvy VCF	1,809	54,882	2,250	2	0.9989	0.9606
C	AgileROH	999	55,891	2,175	98	0.9107	0.9625
C	AUDACITY	1,042	56,462	1,604	55	0.9499	0.9724
C	AutoMAP	1,007	55,711	2,355	90	0.9180	0.9594
C	AutozygosityMapper	960	56,710	1,356	137	0.8751	0.9766
C	H3M2	1,071	53,787	4,279	26	0.9763	0.9263
C	PLINK (VCF)	399	57,098	968	698	0.3637	0.9833
C	Savvy Bam	1,097	54,388	3,678	0	1.0000	0.9367
C	Savvy VCF	1,058	56,184	1,882	39	0.9644	0.9676
D	AgileROH	1,661	57,318	467	68	0.9607	0.9919
D	AUDACITY	1,403	57,534	251	326	0.8115	0.9957
D	AutoMAP	1,639	57,168	617	90	0.9479	0.9893
D	AutozygosityMapper	1,481	57,741	44	248	0.8566	0.9992
D	H3M2	1,638	54,540	3,245	91	0.9474	0.9438
D	PLINK (VCF)	398	57,784	1	1,331	0.2302	1.0000
D	Savvy Bam	1,652	55,121	2,664	77	0.9555	0.9539
D	Savvy VCF	1,646	56,652	1,133	83	0.9520	0.9804
E	AgileROH	4,464	53,982	1,277	79	0.9826	0.9769
E	AUDACITY	3,975	54,655	604	568	0.8750	0.9891
E	AutoMAP	4,217	53,868	1,391	326	0.9282	0.9748
E	AutozygosityMapper	4,412	52,052	3,207	131	0.9712	0.9420
E	H3M2	4,162	51,588	3,671	381	0.9161	0.9336
E	PLINK (VCF)	2,282	55,147	112	2,261	0.5023	0.9980
E	Savvy Bam	4,412	52,052	3,207	131	0.9712	0.9420
E	Savvy VCF	4,315	53,641	1,618	228	0.9498	0.9707
F	AgileROH	8,224	44,926	261	278	0.9673	0.9942
F	AUDACITY	7,261	45,089	98	1,241	0.8540	0.9978
F	AutoMAP	7,946	44,300	887	556	0.9346	0.9804
F	AutozygosityMapper	8,295	41,898	3,289	207	0.9757	0.9272
F	H3M2	8,007	43,019	2,168	495	0.9418	0.9520
F	PLINK (VCF)	3,119	45,176	11	5,383	0.3669	0.9998
F	Savvy Bam	8,295	41,898	3,289	207	0.9757	0.9272
F	Savvy VCF	8,272	44,014	1,173	230	0.9729	0.9740

Supplementary Table S1. Lists of variants found to reside inside and outside of autozygous regions identified by AgileMultiIdeogram , AUDACITY, AutoMAP, AutozygosityMapper, H^3M^2, PLINK using exome variant data were compared to the same variants similarly classified by PLINK using Affymetrix SNP6 genotype data, SavvyHomozygosity and SavvyVCFHomozygosity, to quantify the number of correctly classified variants. These values were used to calculate the true positive rate (TPR) and true negative rate (TNR) for each consanguineous individual's data.

Step by step description of the algorithm

The individual steps of the algorithm that are described below. The process can be split into four distinct sections: steps 1 to 3 filter variants in the dataset, steps 4 to 7 genotype the variants, steps 8 to 10 remove aberrant heterozygous positions, and finally steps 11 and 13 identify autozygous regions. Since the number of variants in a data set varies, the various cutoff values are scaled to suit each data set. This scaling used the parameters X_{filter}, $X_{minimum}$, and X_{call}. Each variant is genotyped using the parameters N_{AA}, N_{BB} and N_{het}.

Abbreviations used:

N_R	The minimum read depth required for a variant to be included
N_{AA}	The minimum proportion of reads for the reference allele required for a variant to be call homozygous reference
N_{BB}	The maximum proportion of reads for the reference allele required for a variant to be call homozygous non-reference.
N_{het}	The degree of divergence from 0.5 that the proportion of reads for the reference allele required for a variant to be called heterozygous (i.e., heterozygous if in the range $0.5 \pm N_{het}$)
X_{filter}	This constant is used to determine the minimum number of flanking homozygous variants before a heterozygous variant is ignored. After optimisation of the algorithm, X_{filter} has a value of 386.
$X_{minimum}$	This constant is used to determine the minimum number of homozygous variant that a heterozygous variant can be from another heterozygous variant before it is ignored. After optimisation, $X_{minimum}$ has a value of 0.1.
X_{call}	This constant is used to determine the minimum length a run of homozygous variants required for them to be set as autozygous. Its value is 575.

Variant filtering

1. Variants without an RS ID are discounted (optional for exome data)
2. Variants with a read depth below minimum read depth (N_R) are discounted.
3. Variants with more than two alleles are discounted.

Genotyping variants

4. A variant is deemed homozygous for the non-reference allele if the proportion of reads representing this allele is above the cutoff ($1 – N_{BB}$).

5. Otherwise, a variant is deemed heterozygous if the proportion of reads representing the reference allele lies in the range 0.5 ± N_{het}.
6. Otherwise, a variant is deemed homozygous for the reference allele if the proportion of reads representing the reference allele is above N_{AA}.
7. Variant that did not receive a genotype are ignored.

Removal of aberrant heterozygous variants

8. Heterozygous variants are discounted when flanked by N_{filter} homozygous variants, where N_{filter} = (variants on chr1 × X_{filter}) / 70700
9. and is more than N_{gap} variants from the nearest heterozygous variant where $N_{gap} = N_{filter} \times X_{minimum}$
10. Steps 7 to 8 are repeated twice, for a total of three iterations.

Identifying autozygous variants

11. Autozygous regions are called if they consist of more than N_{call} variants, where: N_{call} = (variants on chr1 × X_{call}) / 70700
12. If an autozygous region is less than 500 Kb in length, it is discounted
13. If a non-autozygous region is shorter than 100 Kb and flanked by two autozygous regions, it and the flanking regions are merged into one autozygous region

Steps 7 through 10 use cut-off values linked to the size of the variant data sets next section contains a walk-through description of how these values are generated for a hypothetical data set containing 3,000 SNPs on chromosome 1.

Detection of homozygous regions, based on the presence of 3,000 variants on Chromosome 1

The process by which autozygous regions are detected is covered in steps 7 to 10 of the workflow described in the main text, with the values of the parameters shown in Table 1. These steps depend on the values of N_{filter}, N_{gap} and N_{call}, which in turn depend on the number of variants in the dataset

located on Chromosome 1. In this example, if the latter value is 3,000, the value of N_{filter} is determined as 3,000 × X_{filter} / 70,700; since X_{filter} is 386, the value of N_{filter} is 3,000 × 386 / 70,700 = 16 (rounded to the nearest whole number) [step 7]. Similarly, N_{gap} is given as N_{filter} × $X_{minimum}$; as $X_{minimum}$ is 0.1, N_{gap} is 16 × 0.1 = 2 (rounded to the nearest whole number) [step 8]. Consequently, if a heterozygous variant is flanked by more than 16 homozygous variants and is more than 2 homozygous variants from the nearest heterozygous variant, it will be discounted. The length of the homozygous runs is recalculated, and the process is repeated two more times [step 9]. Once the putatively erroneous heterozygous variants have been removed, any run of homozygous variants over N_{call} is reported as an autozygous region, where N_{call} = 3,000 × X_{call} / 70,700; therefore N_{call} = 3,000 × 575 / 70,700= 24 (rounded to the nearest whole number).

Figures

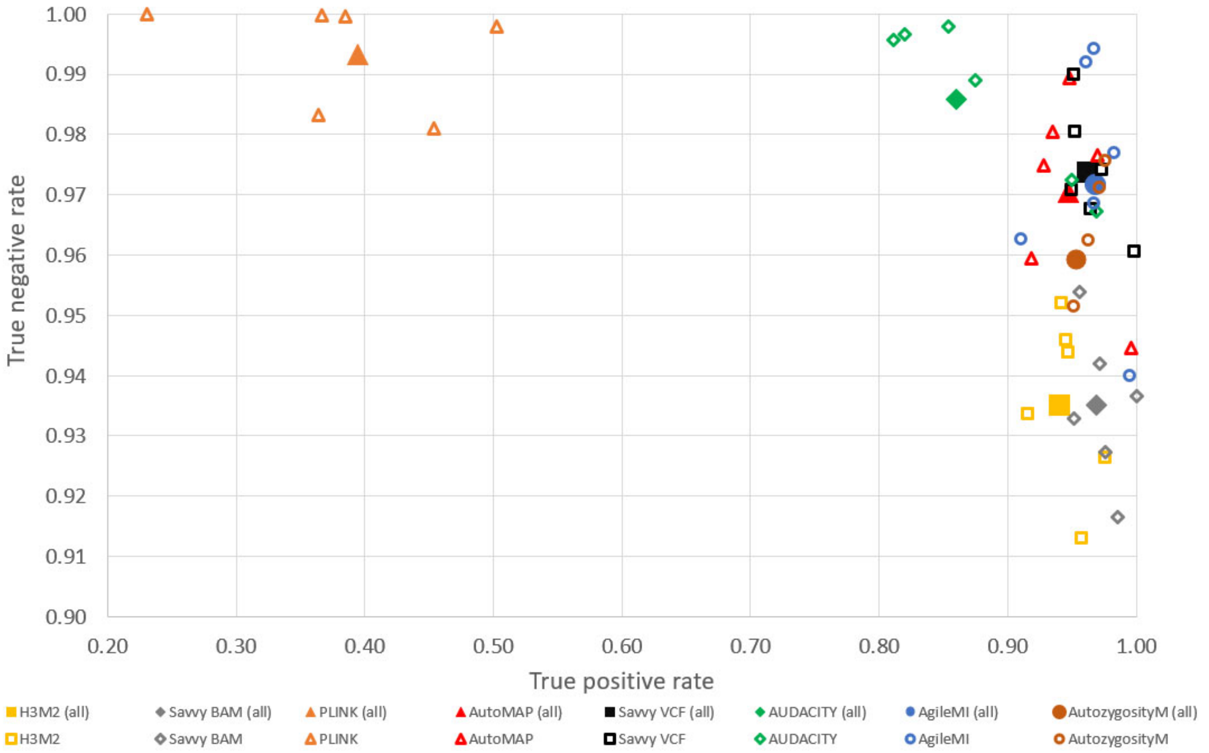

Supplementary Figure S1. Graph demonstrating the true positive and true negative rates of variant classification using autozygous regions identified by AgileMultiIdeogram (blue triangle), AutoMAP (red circle), H^3M^2 (yellow square), SavvyHomozygous (grey diamond), SavvyVCFHomozygous (black square), AutozygosityMapper (purple circle) and PLINK (orange triangle) using exome data. The larger solid symbols represent the aggregate values for the 6 samples, while the smaller symbol outlines represent the individual scores.

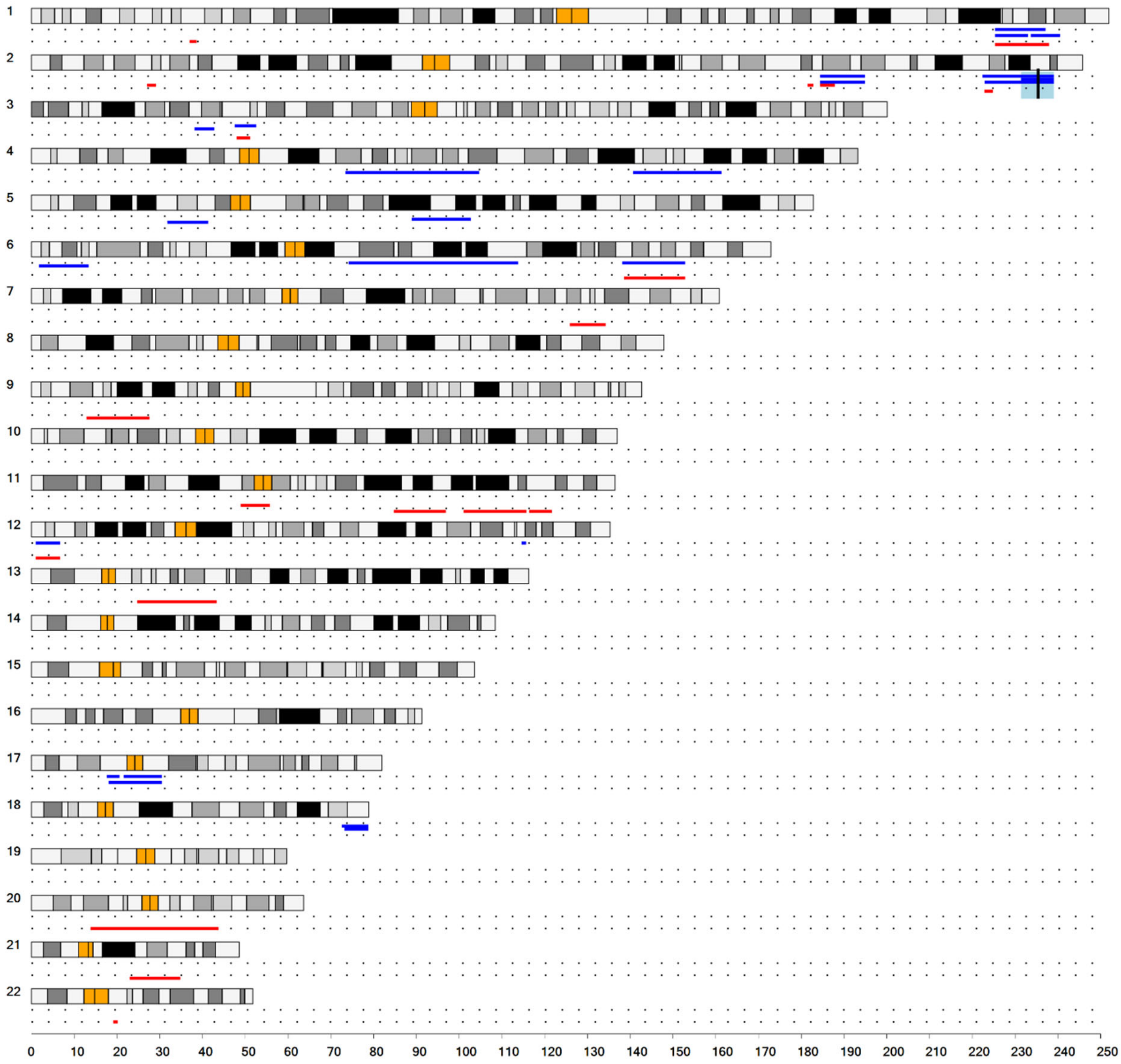

Supplementary Figure S2. Aligned autozygous regions in a family of four siblings and their parents, in which three siblings are affected by the lethal multiple pterygium syndrome (MIM 253290). Autozygous regions in affected and unaffected individuals are shown as blue and red horizontal lines respectively. The pale blue rectangle indicates autozygous regions common to all affected individuals, and the vertical black line marks the position of the *CHRNG* gene.

Supplementary Figure S3. Comparison of autozygous regions identified by AgileMultiIdeogram using whole exome sequencing variant data (dark blue segments in outer circle) and Affymetrix SNP6 microarray SNP genotype data (pale red segments in inner circle). Figures A to R show the samples used in the training dataset (sample R has no autozygous regions). Figures S to X show samples used in the testing data set. The order of the images

A: Training sample

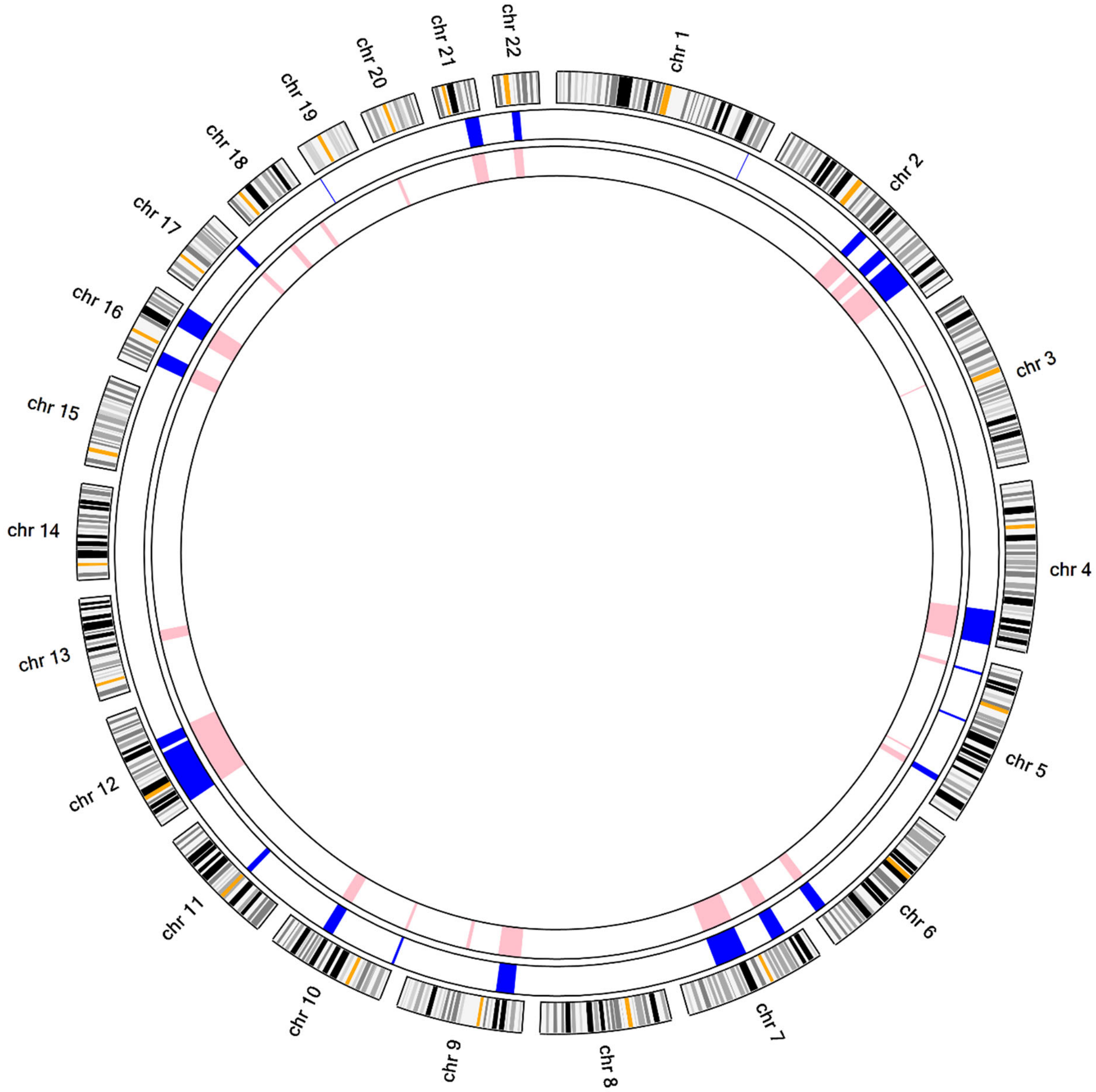

B: **Training sample**

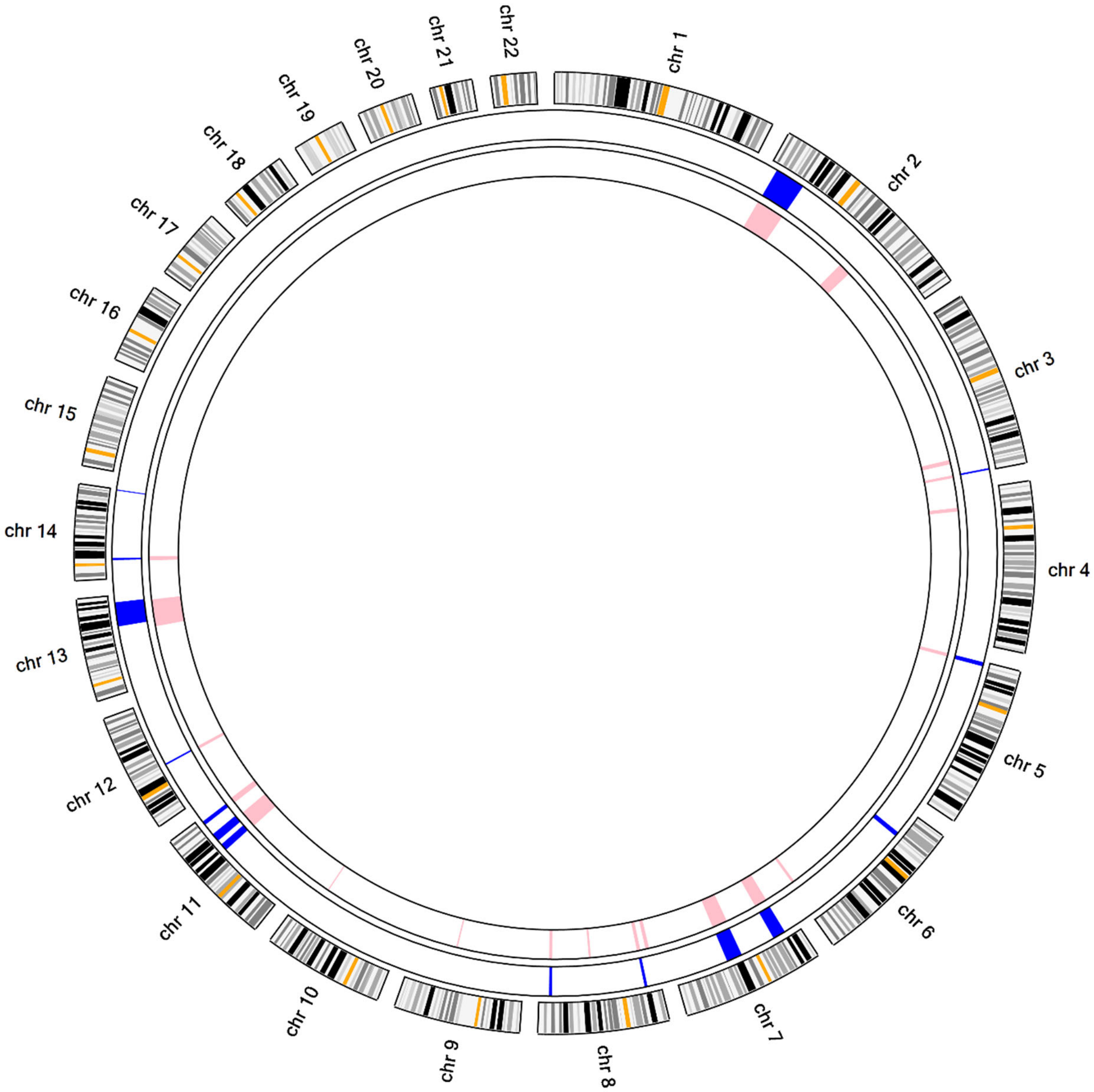

C: **Training sample**

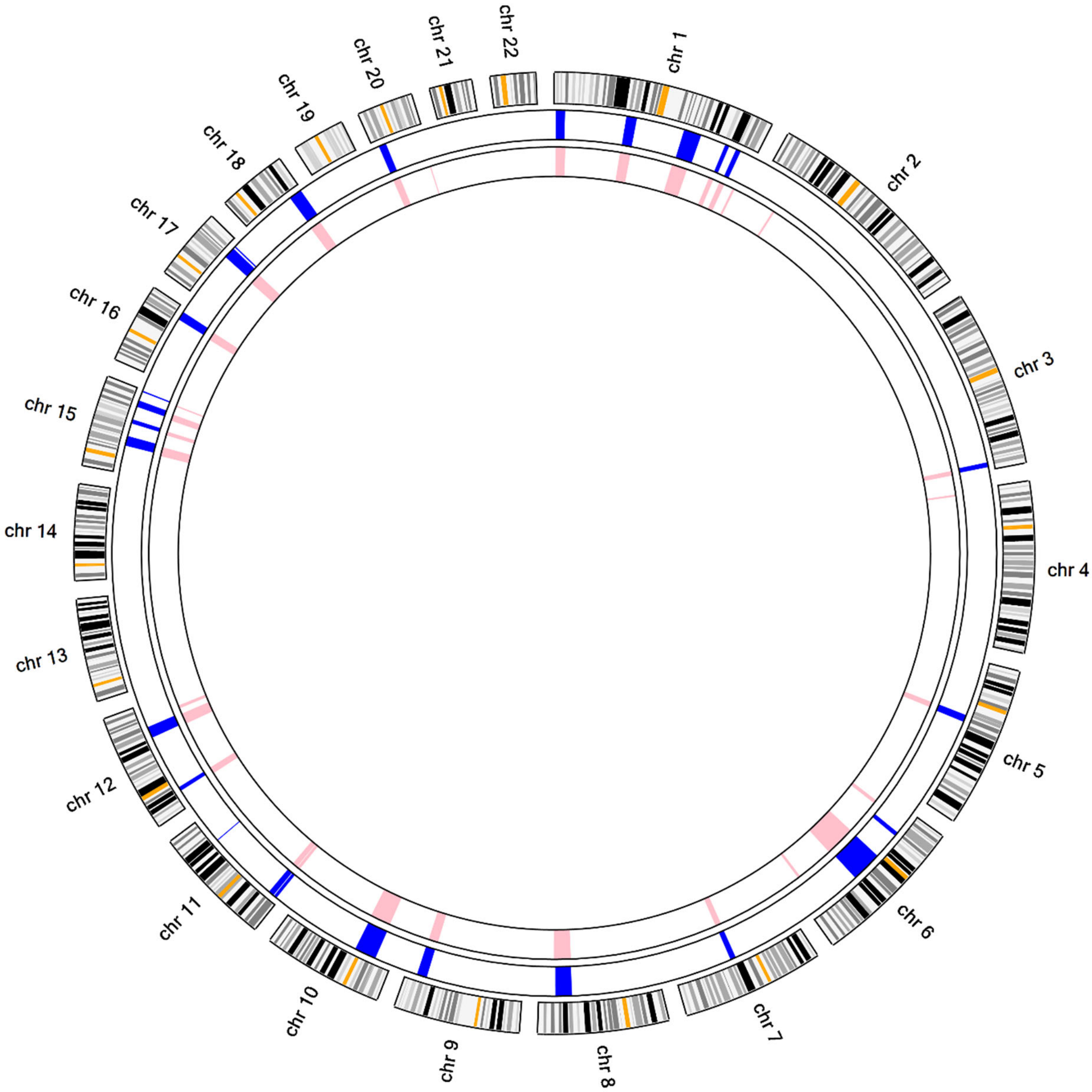

D**: Training sample**

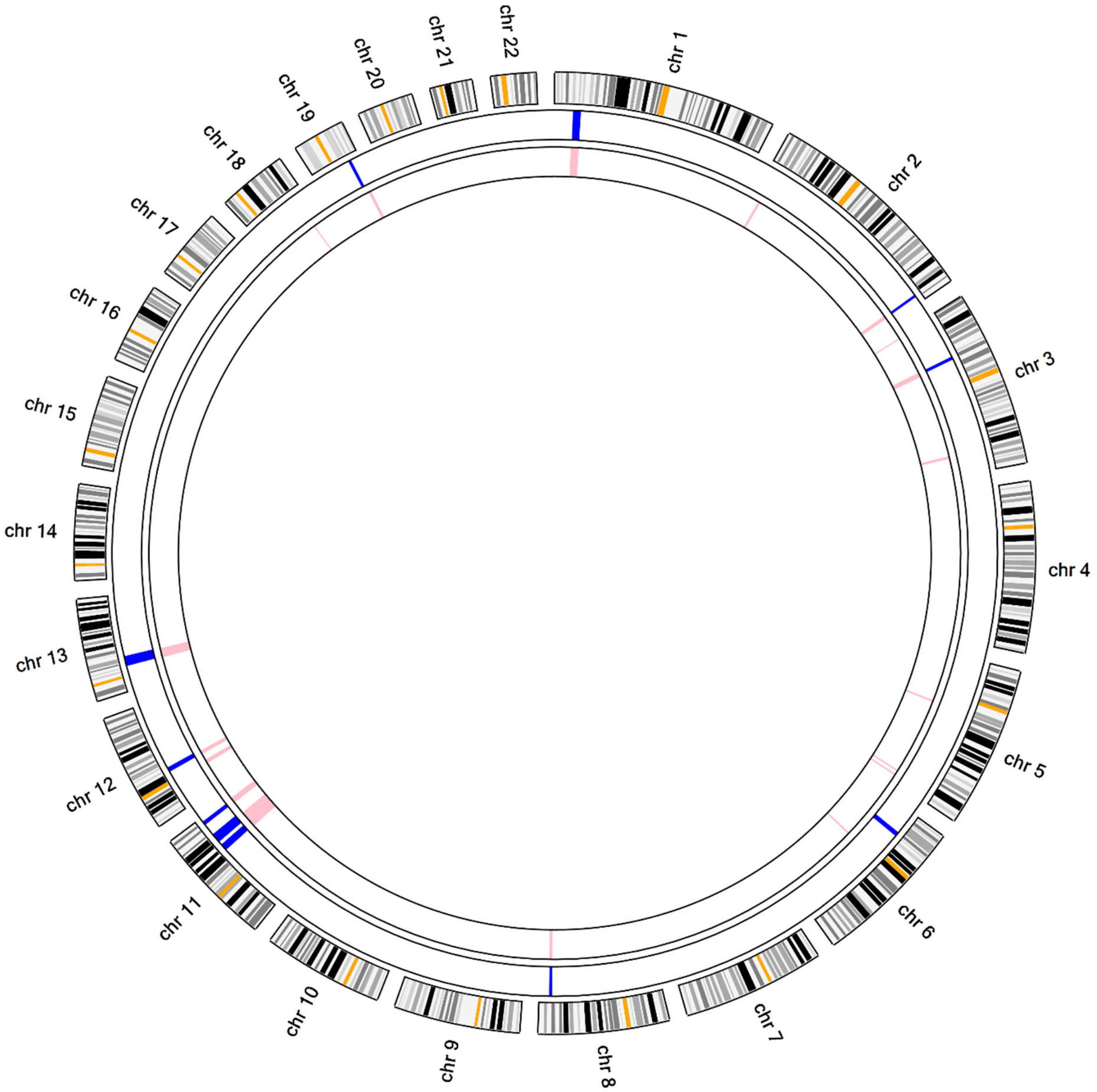

E: **Training sample**

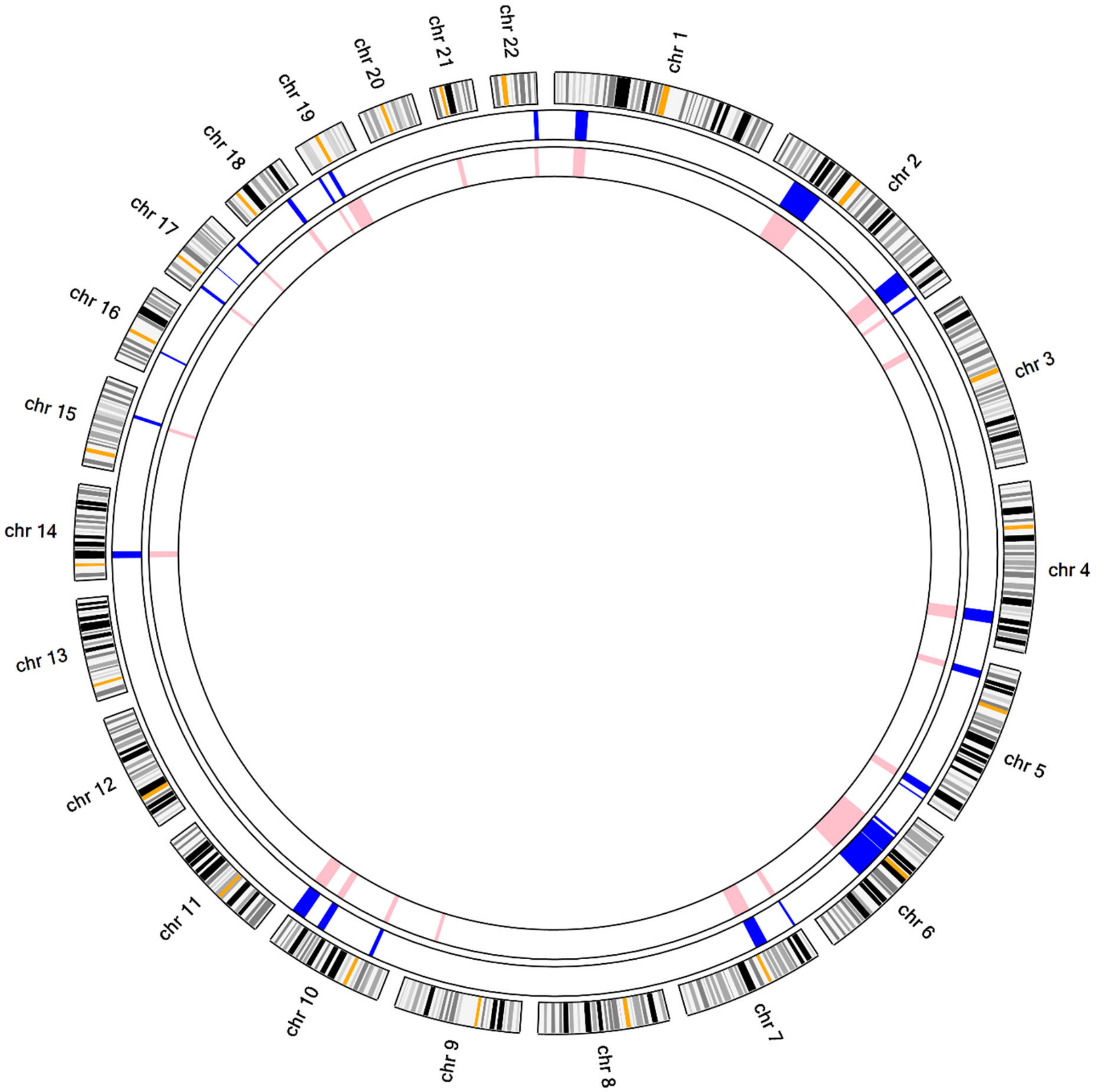

F**: Training sample**

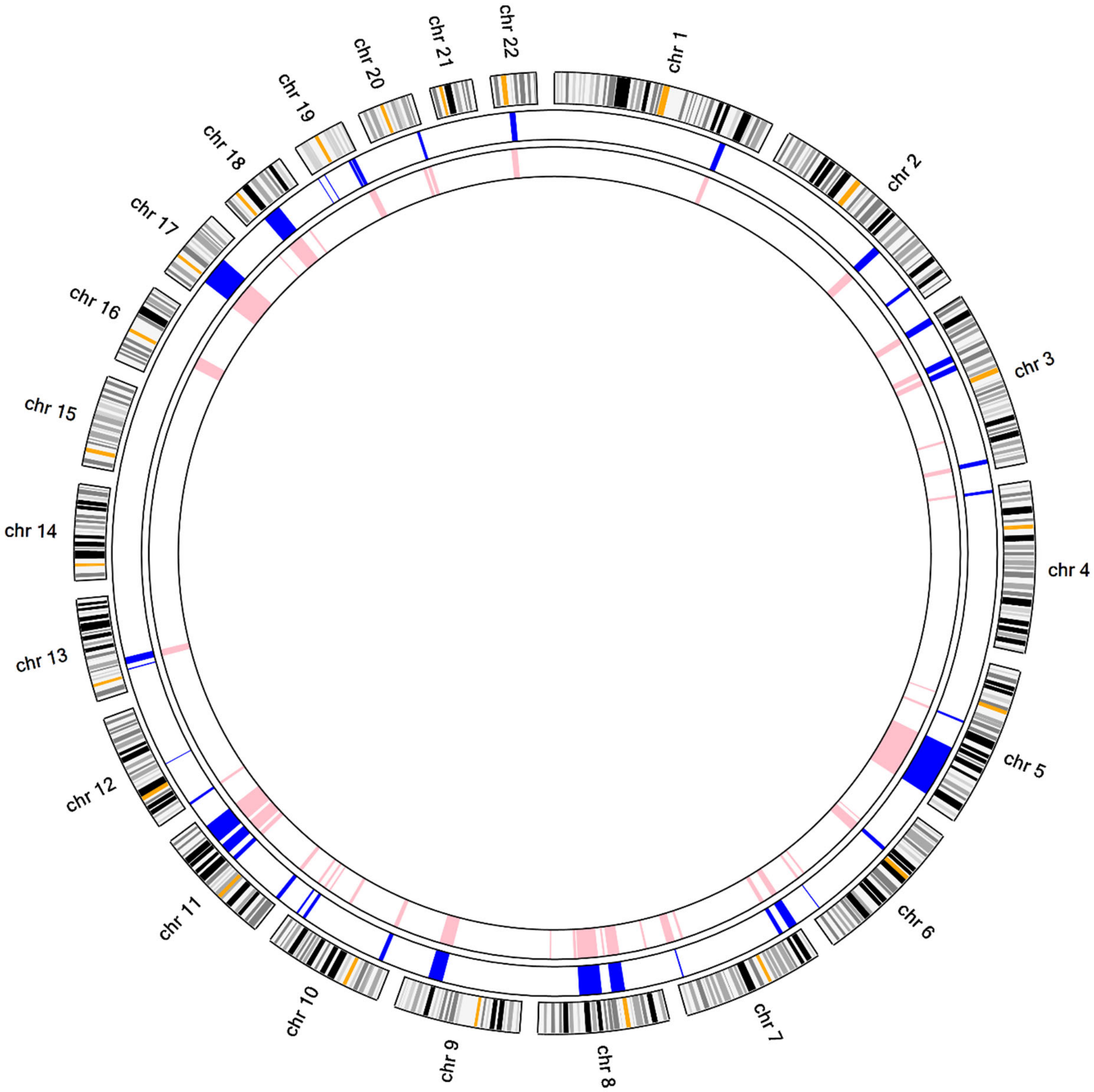

G**: Training sample**

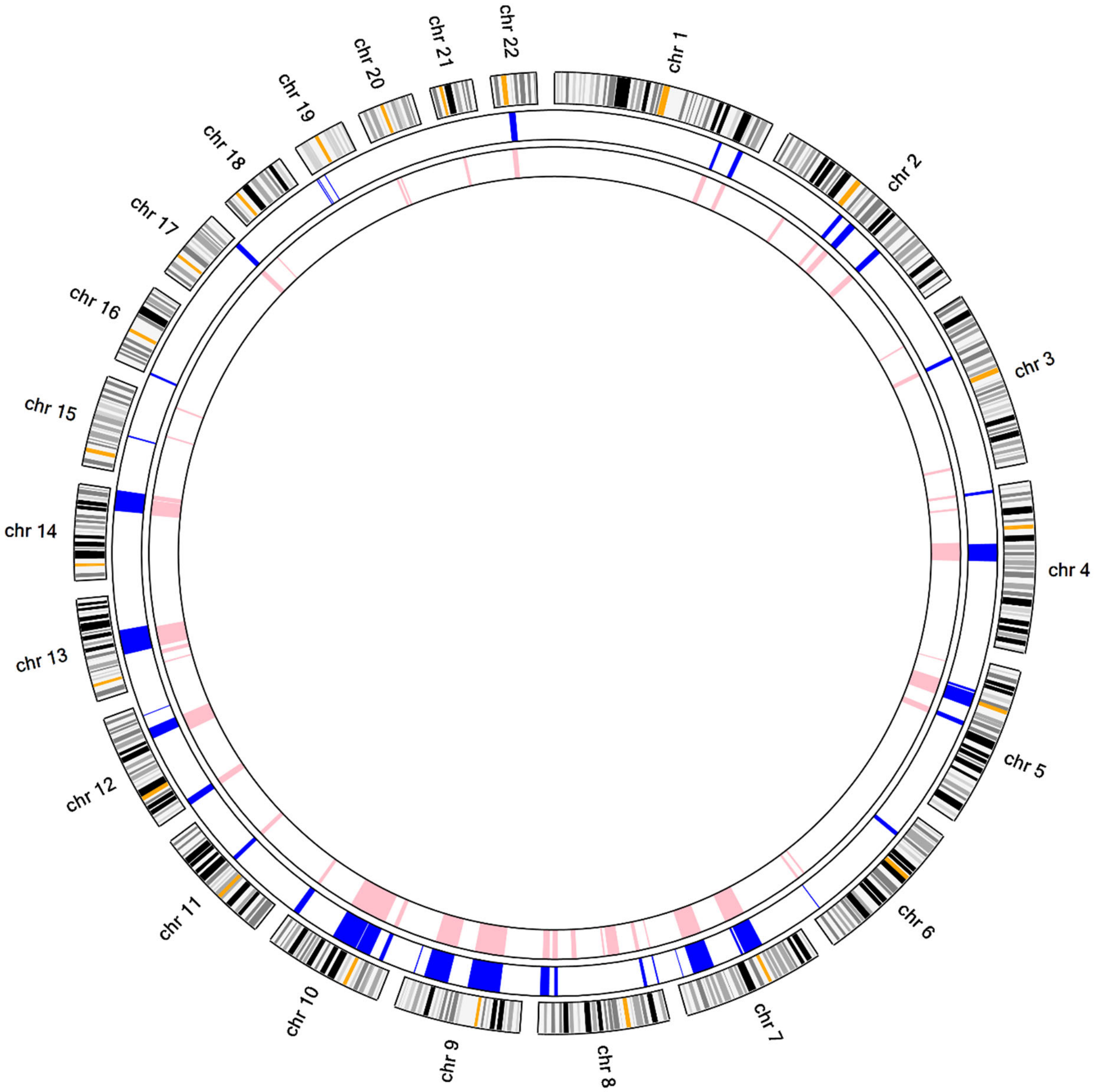

H: **Training sample**

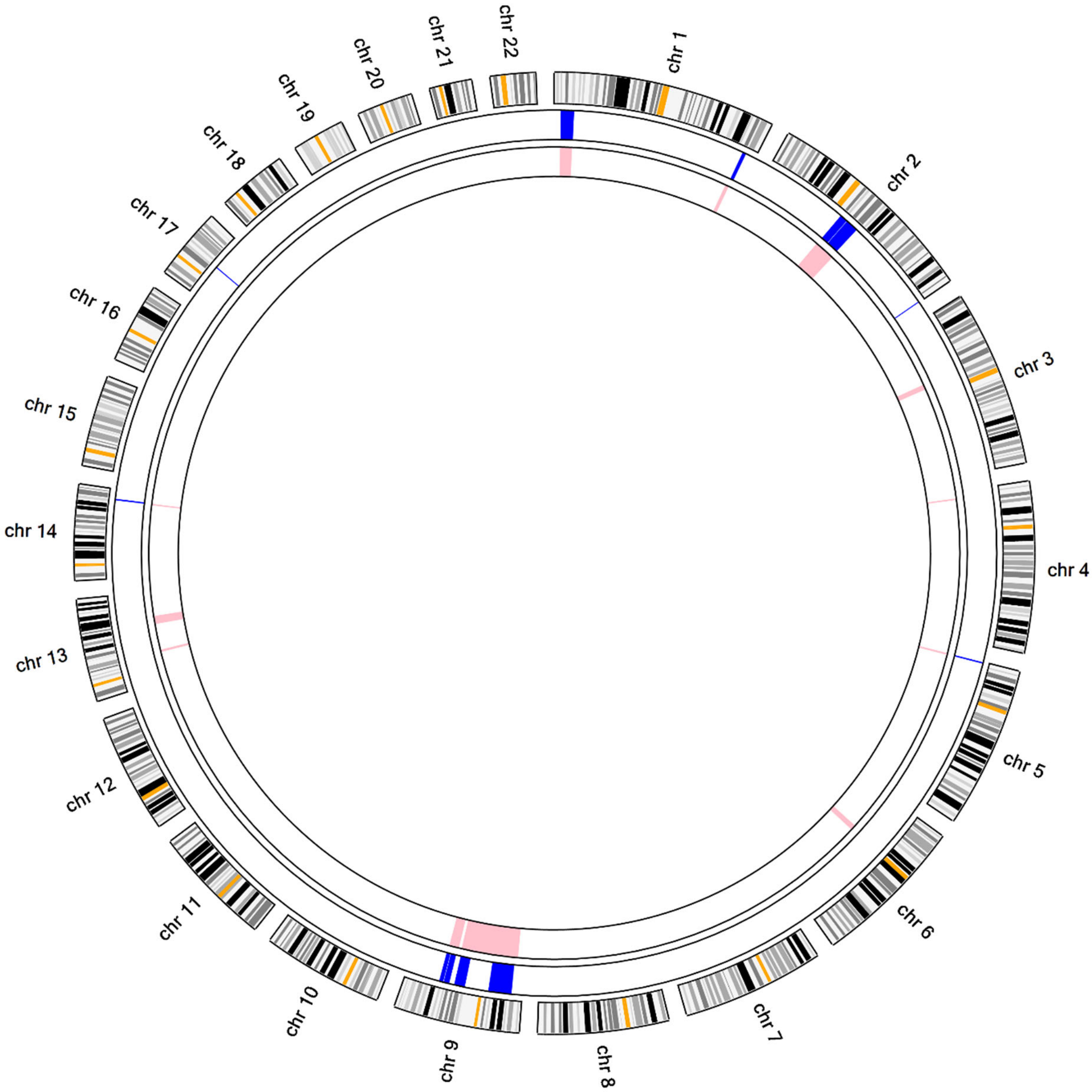

I: Training sample

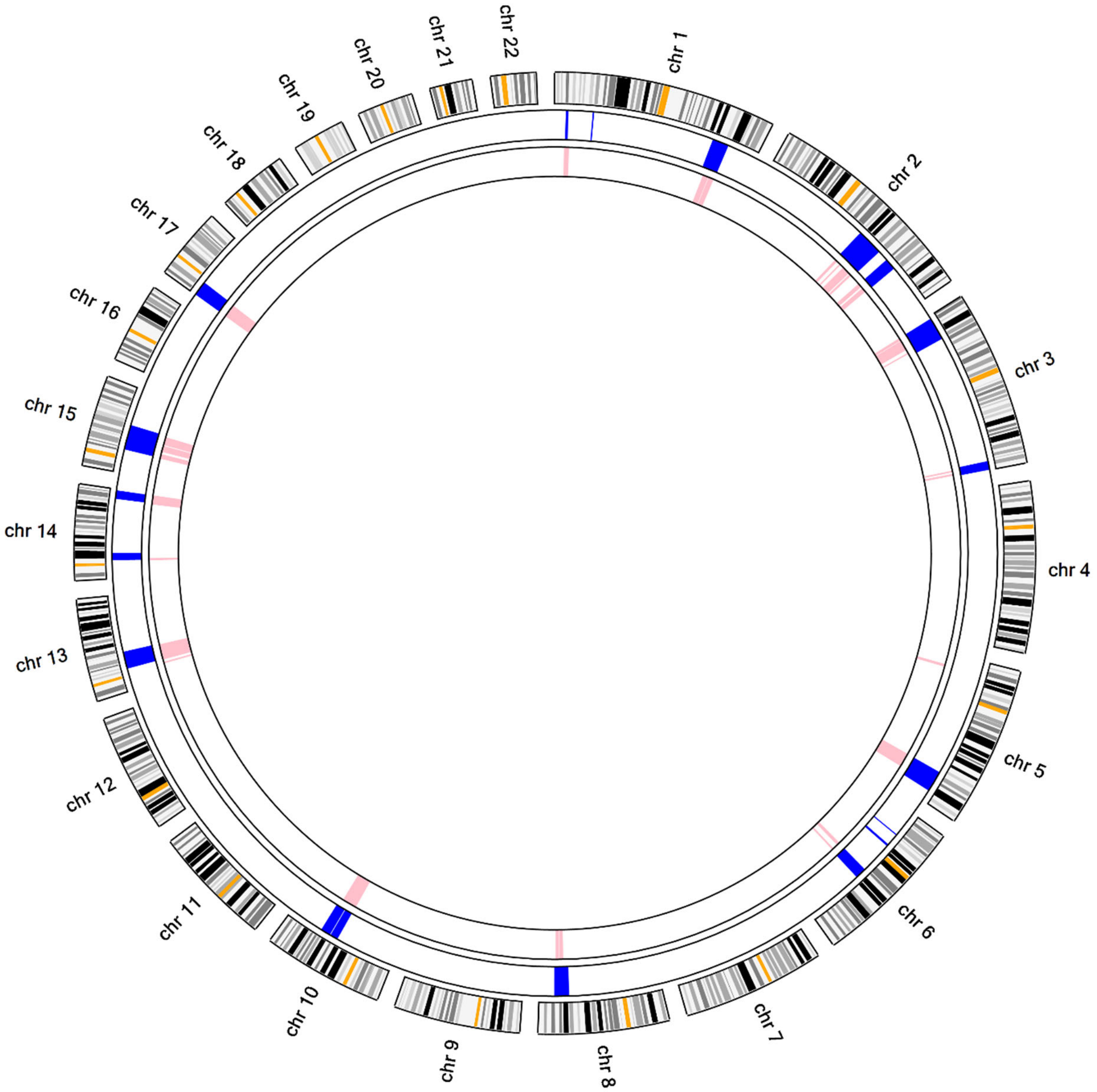

J: Training sample

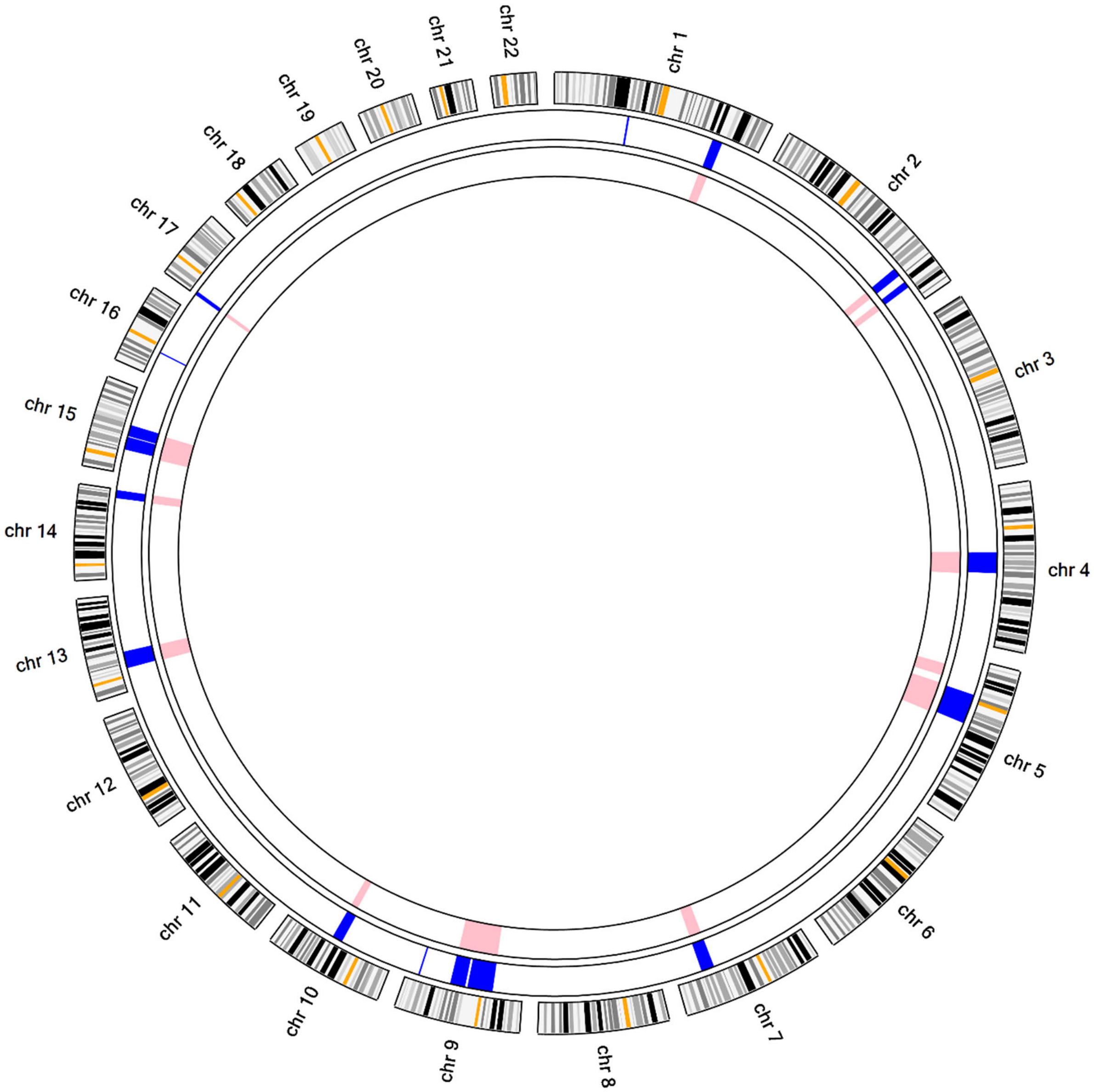

K: **Training sample**

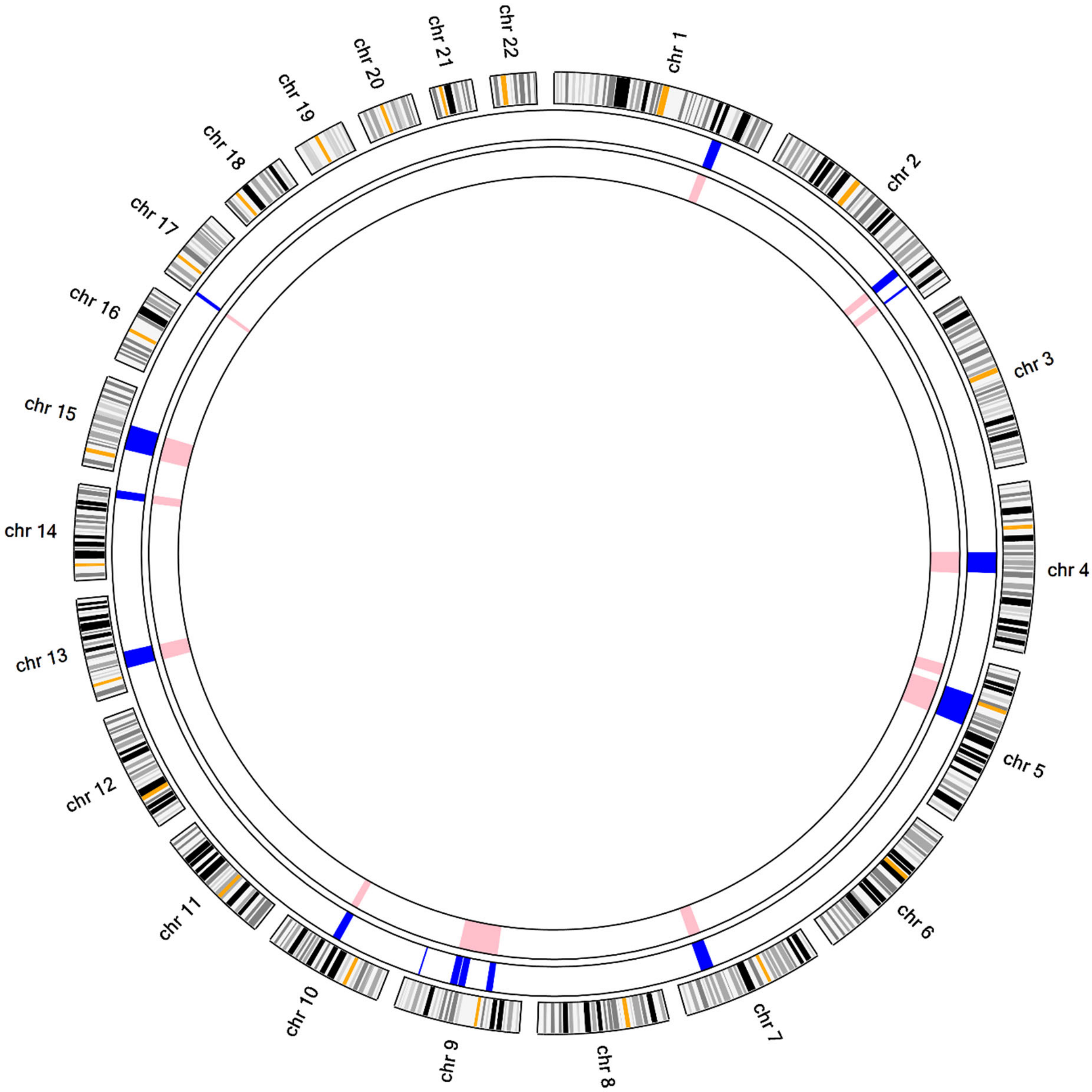

L: **Training sample**

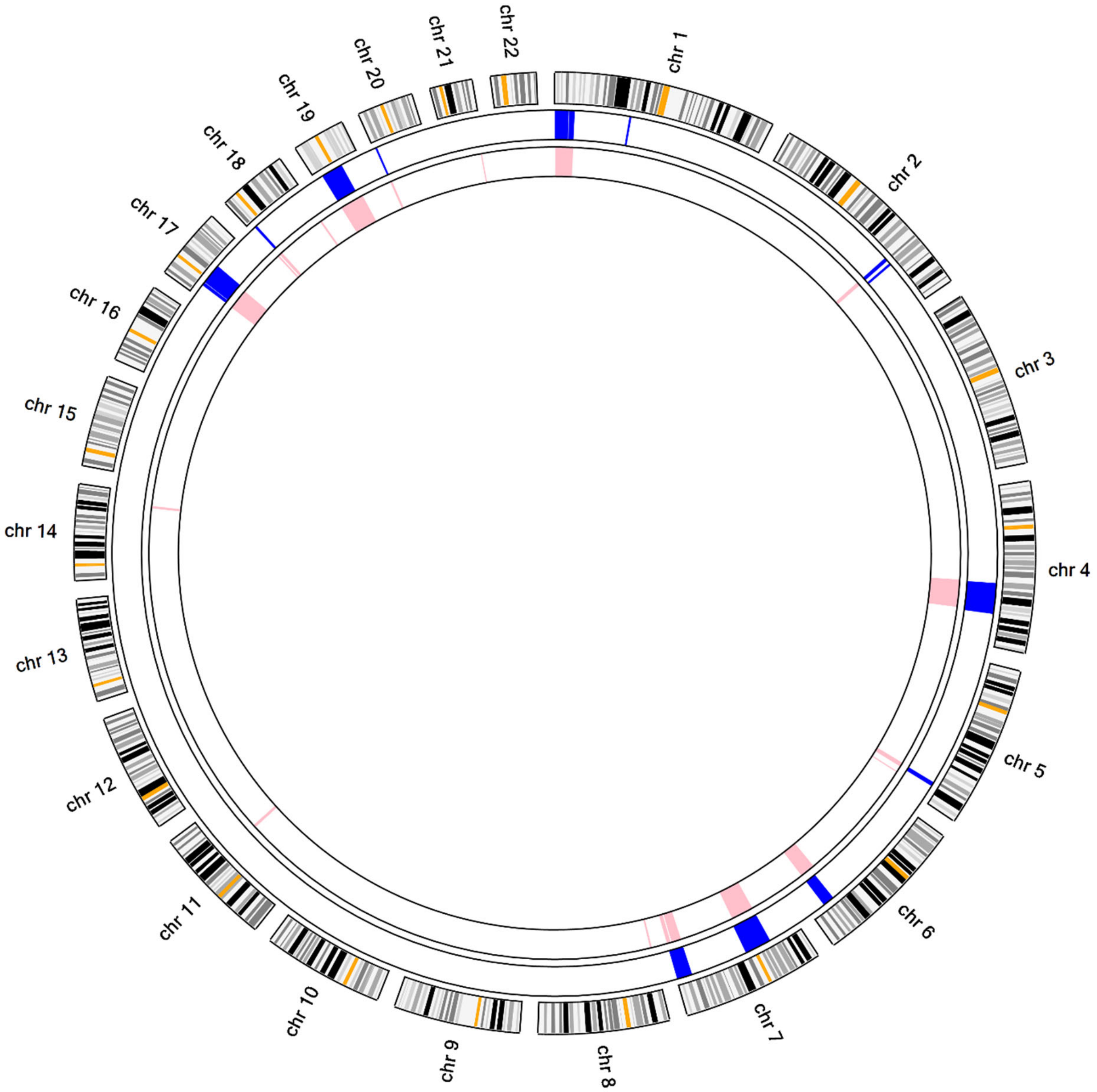

M**: Training sample**

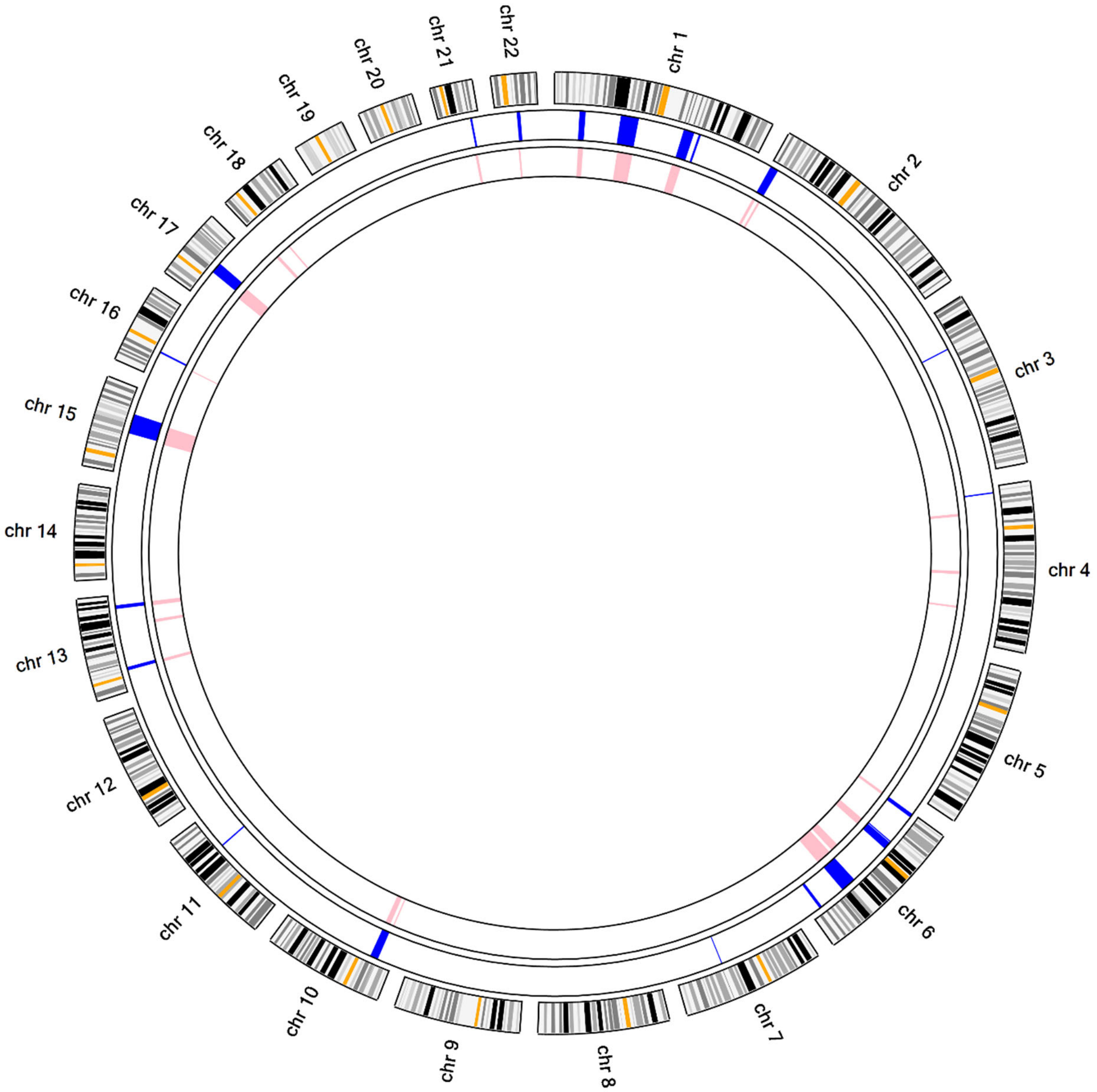

N: **Training sample**

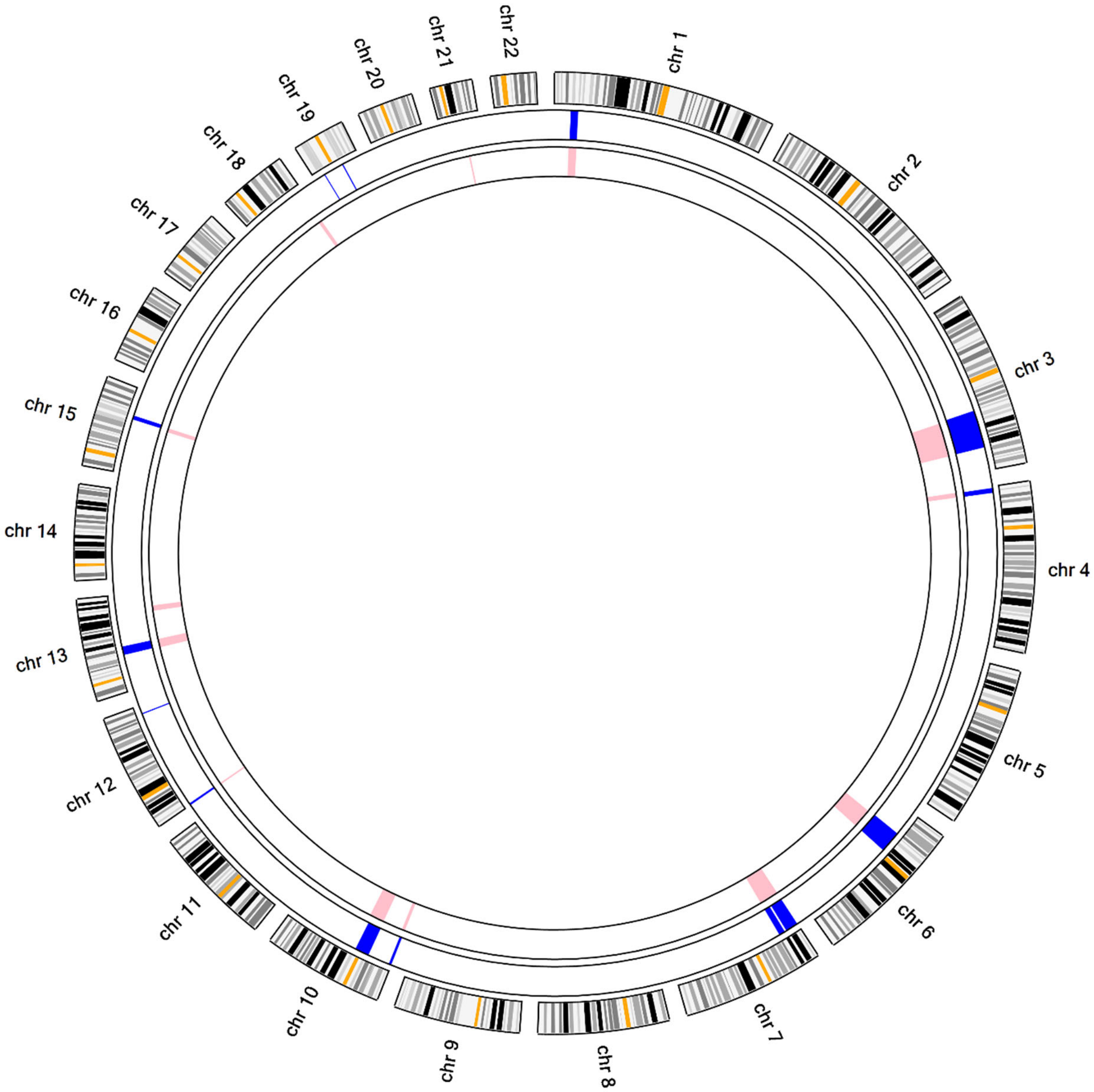

O: **Training sample**

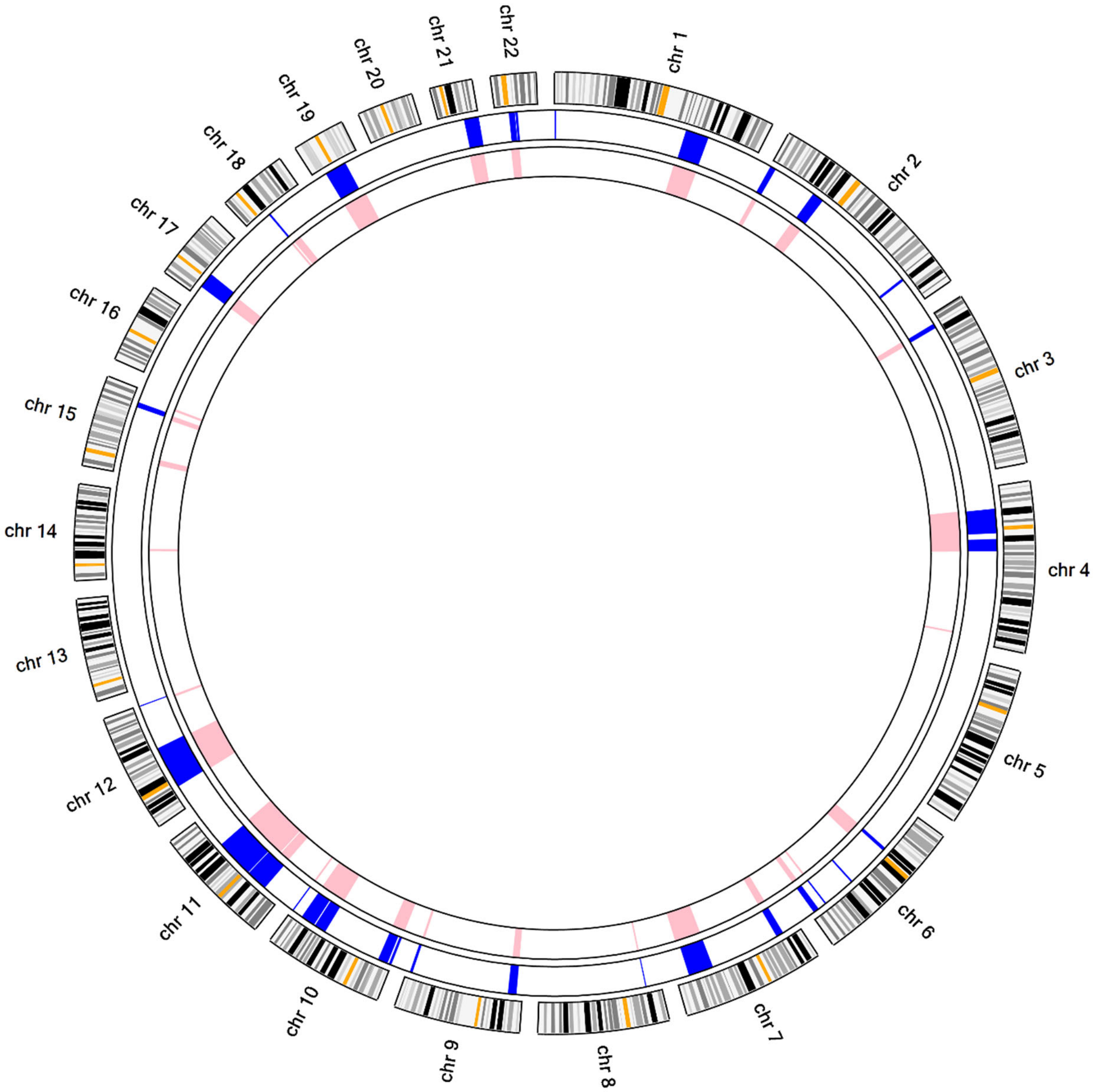

P: **Training sample**

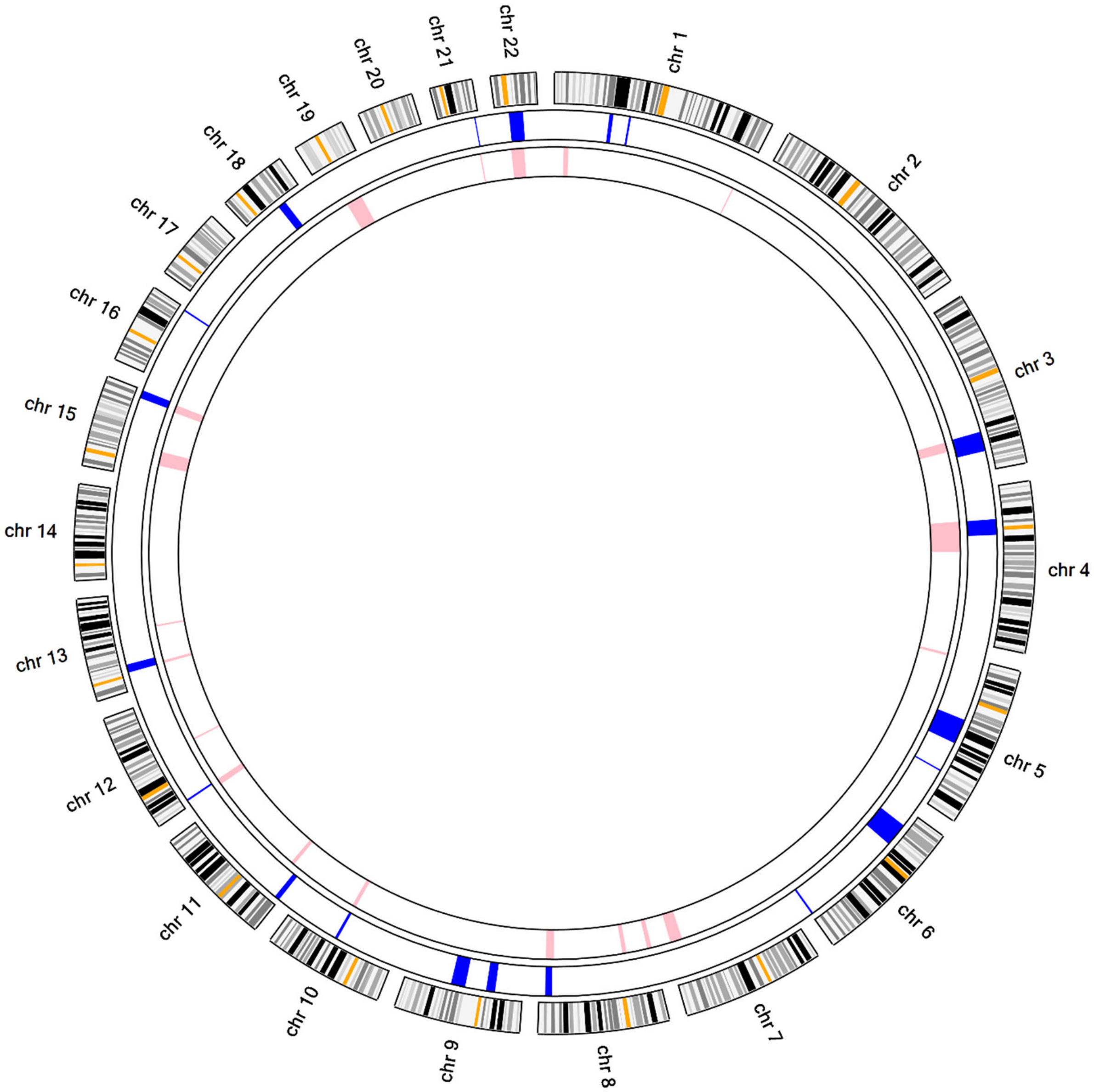

Q: **Training sample**

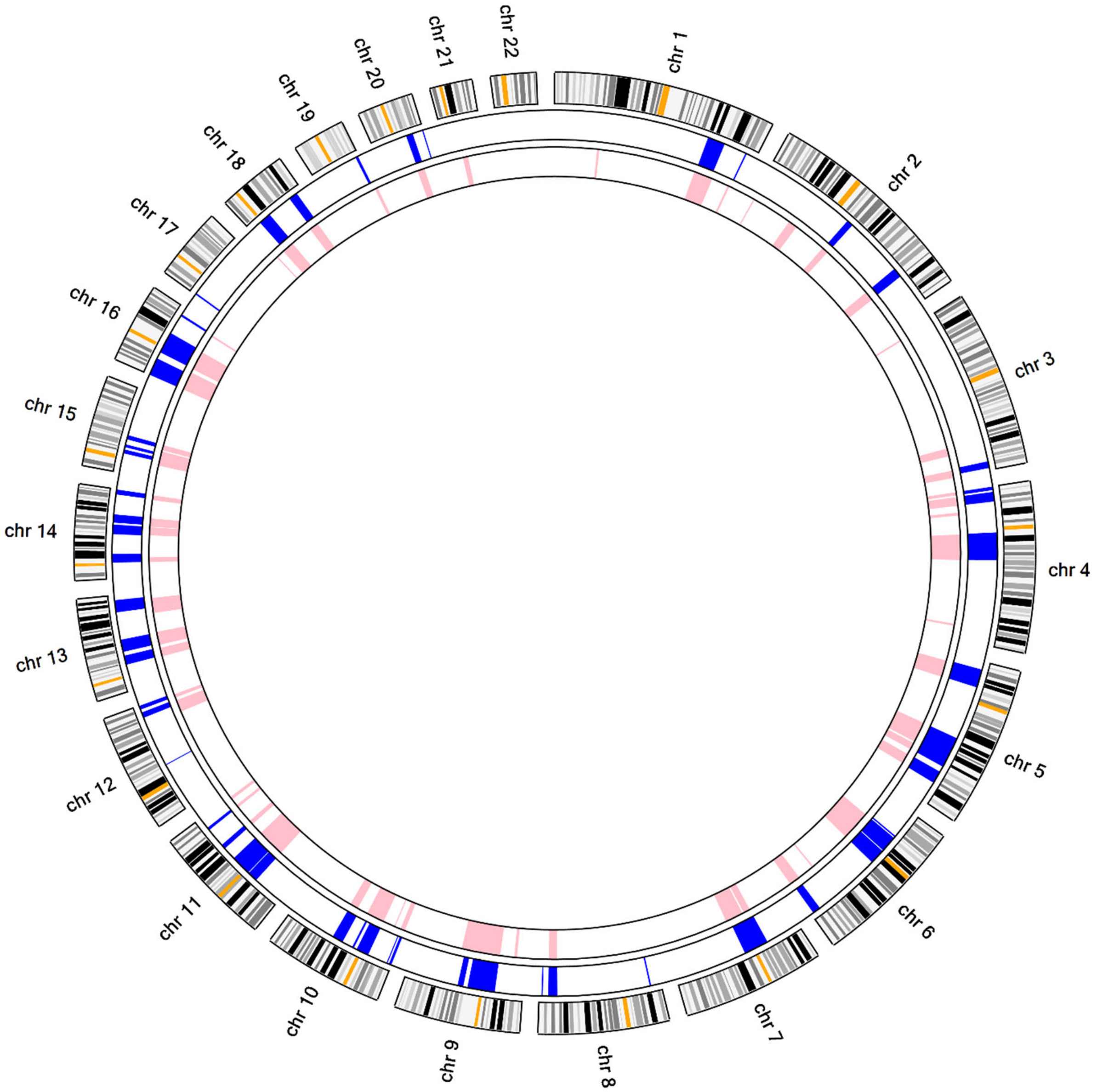

R: **Training sample**

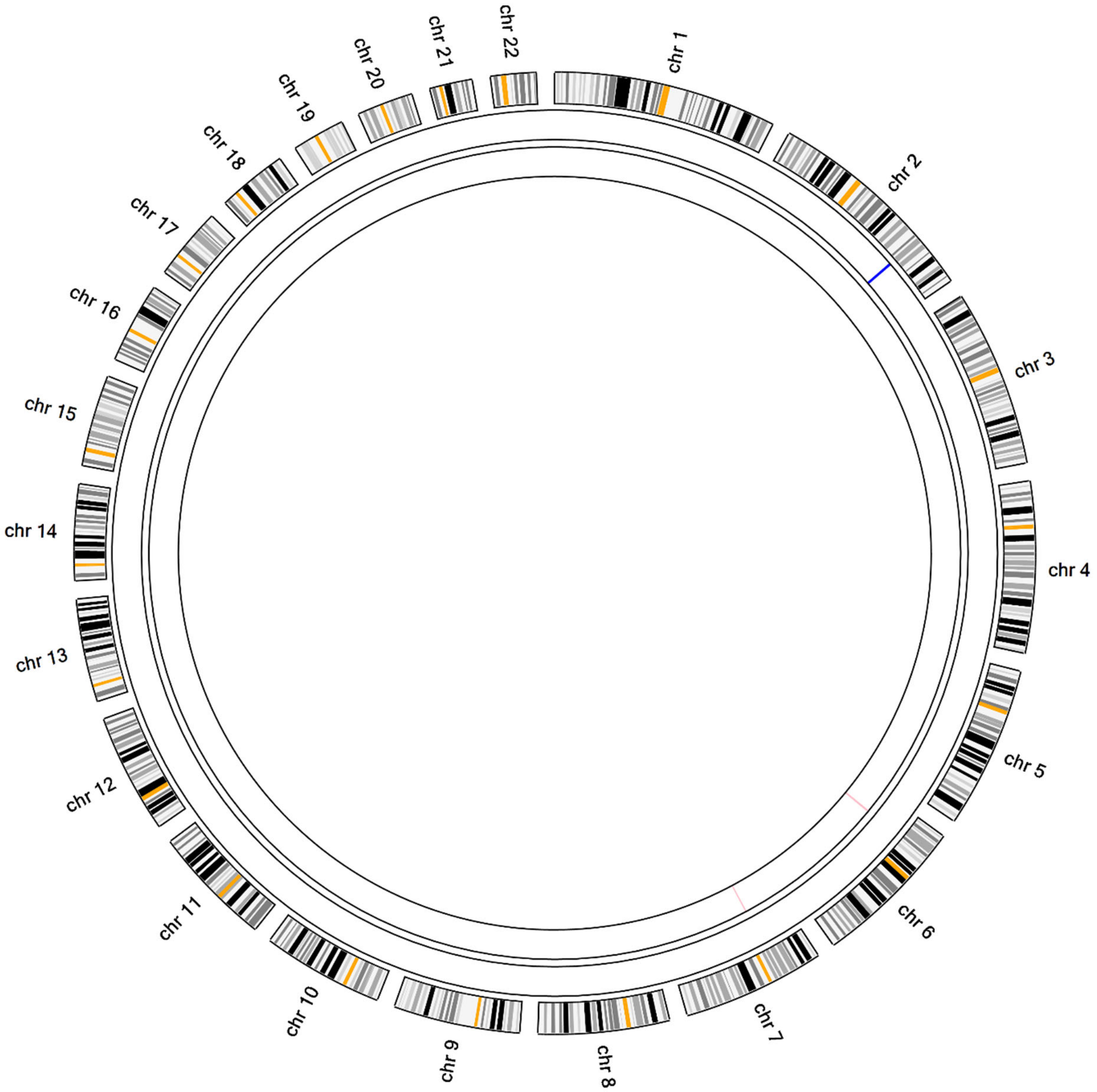

S: **Testing sample**

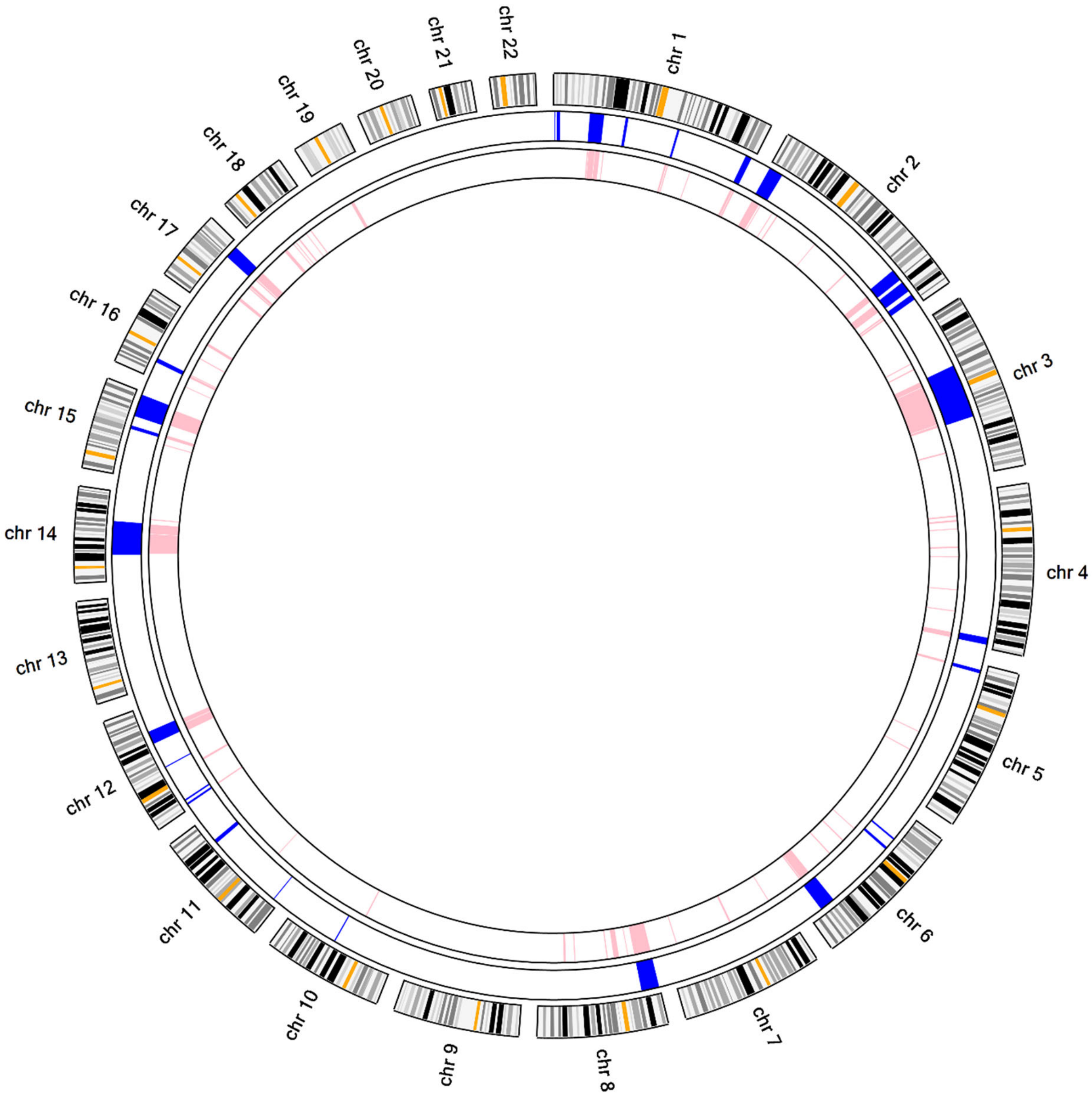

T: **Testing sample**

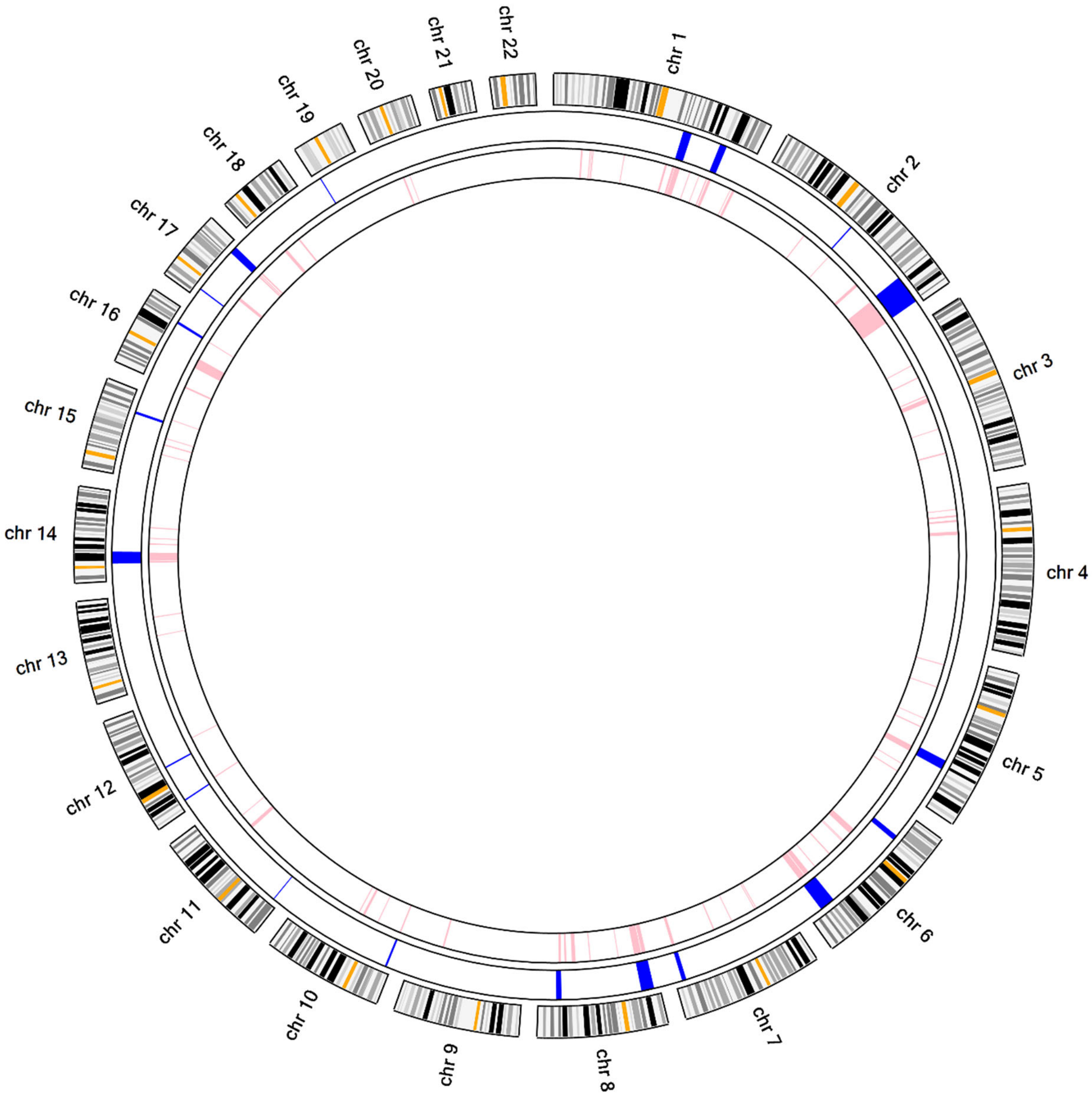

U: **Testing sample**

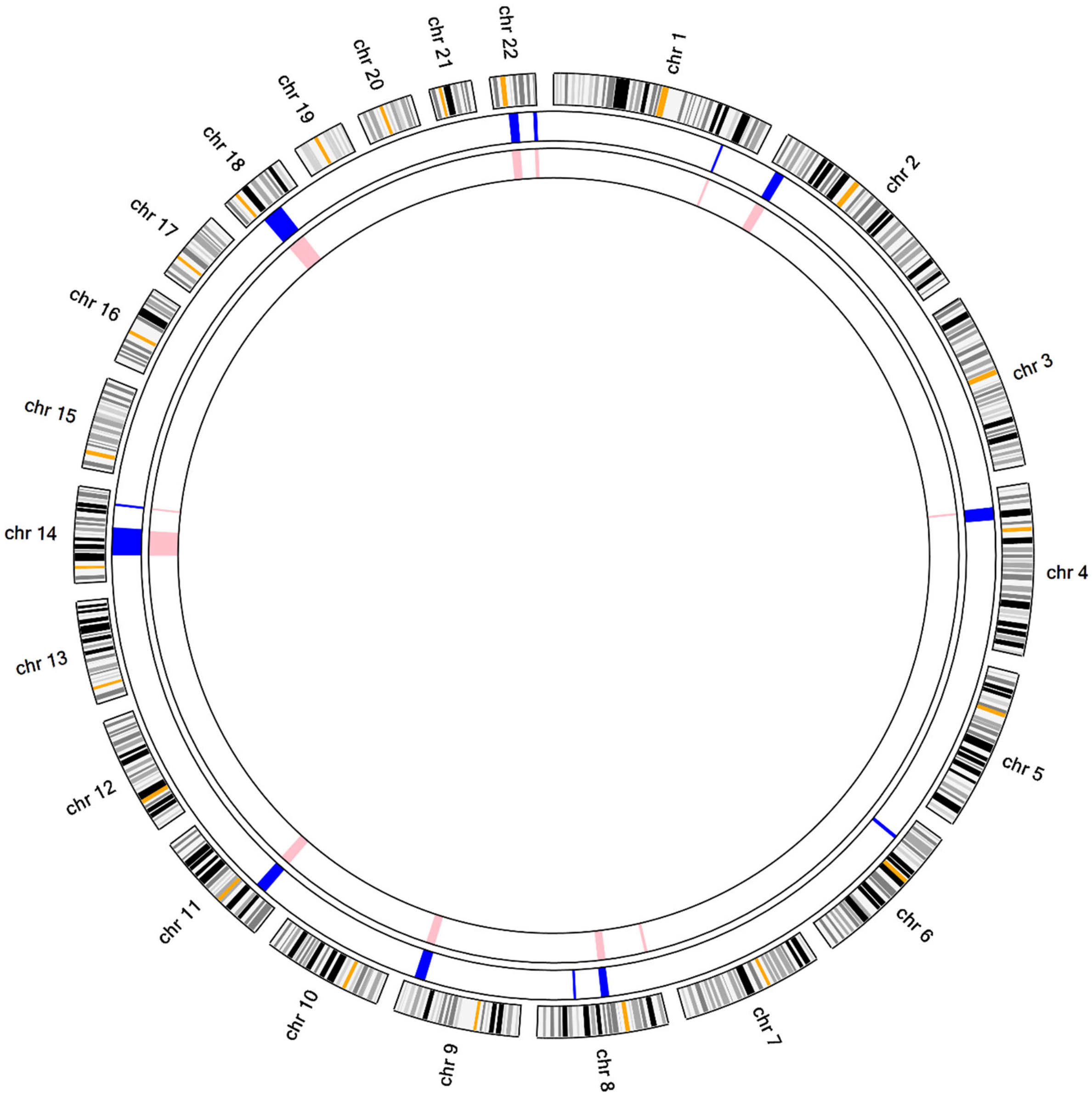

V: **Testing sample**

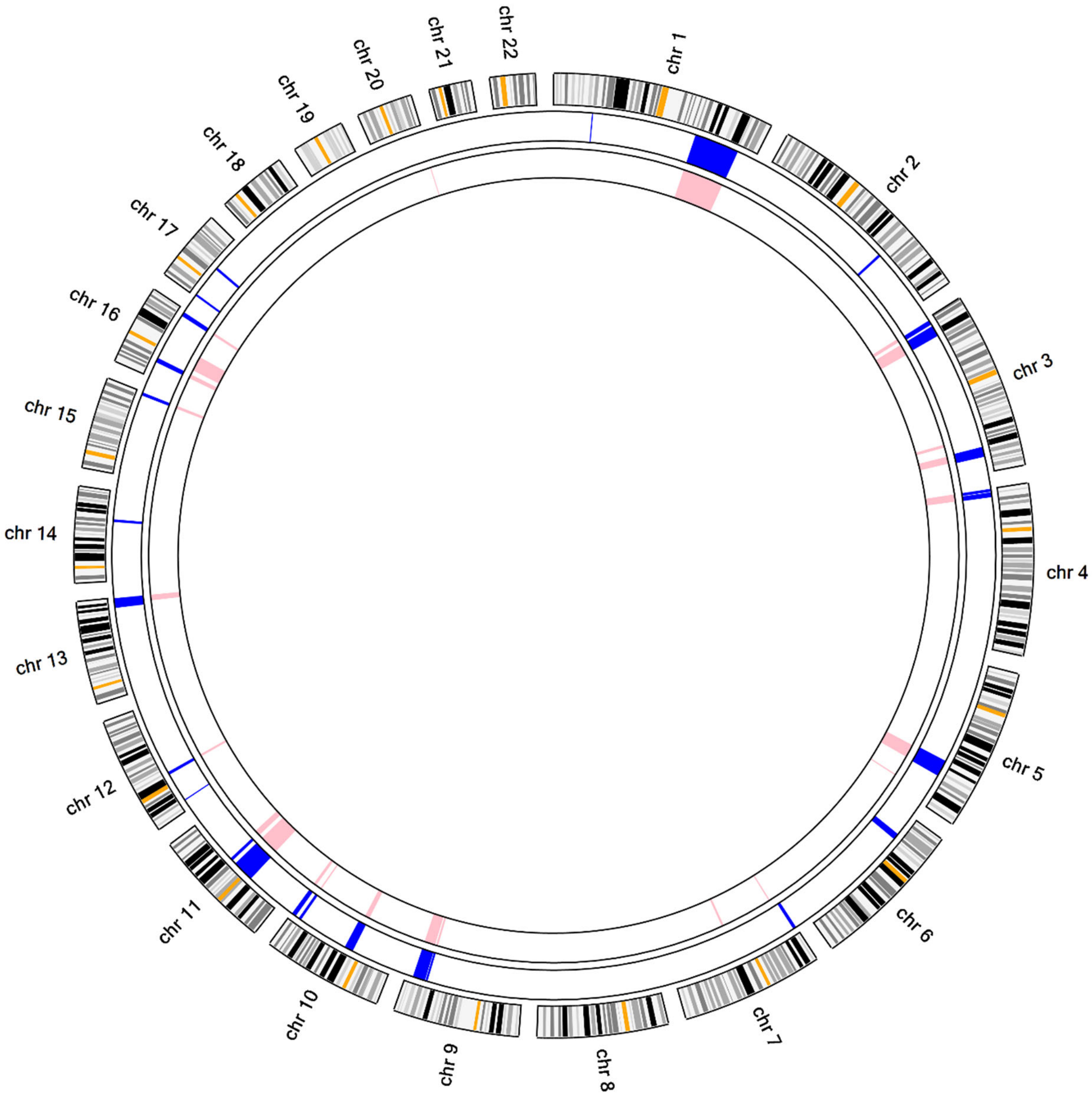

W: **Testing sample**

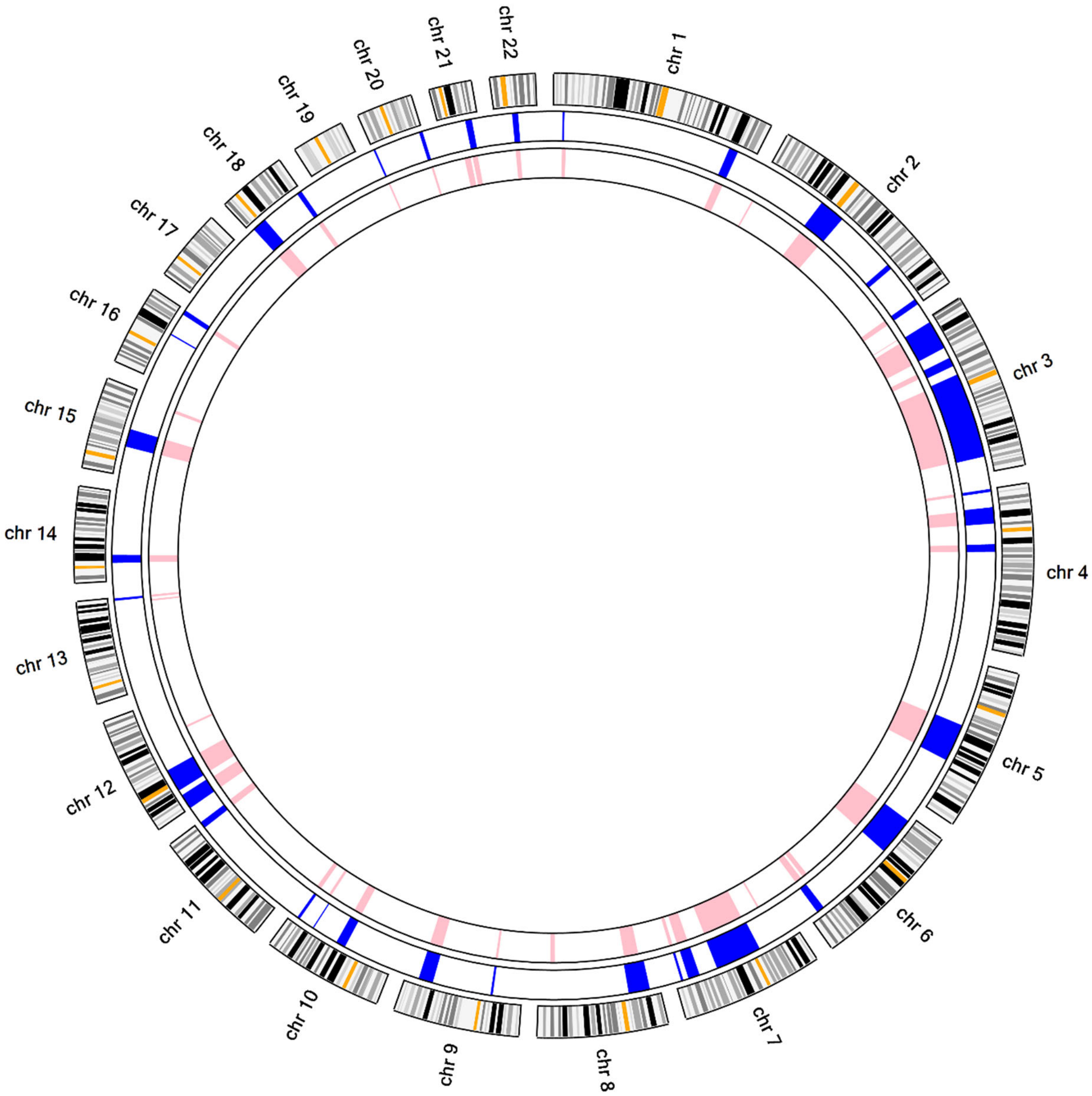

X: **Testing sample**

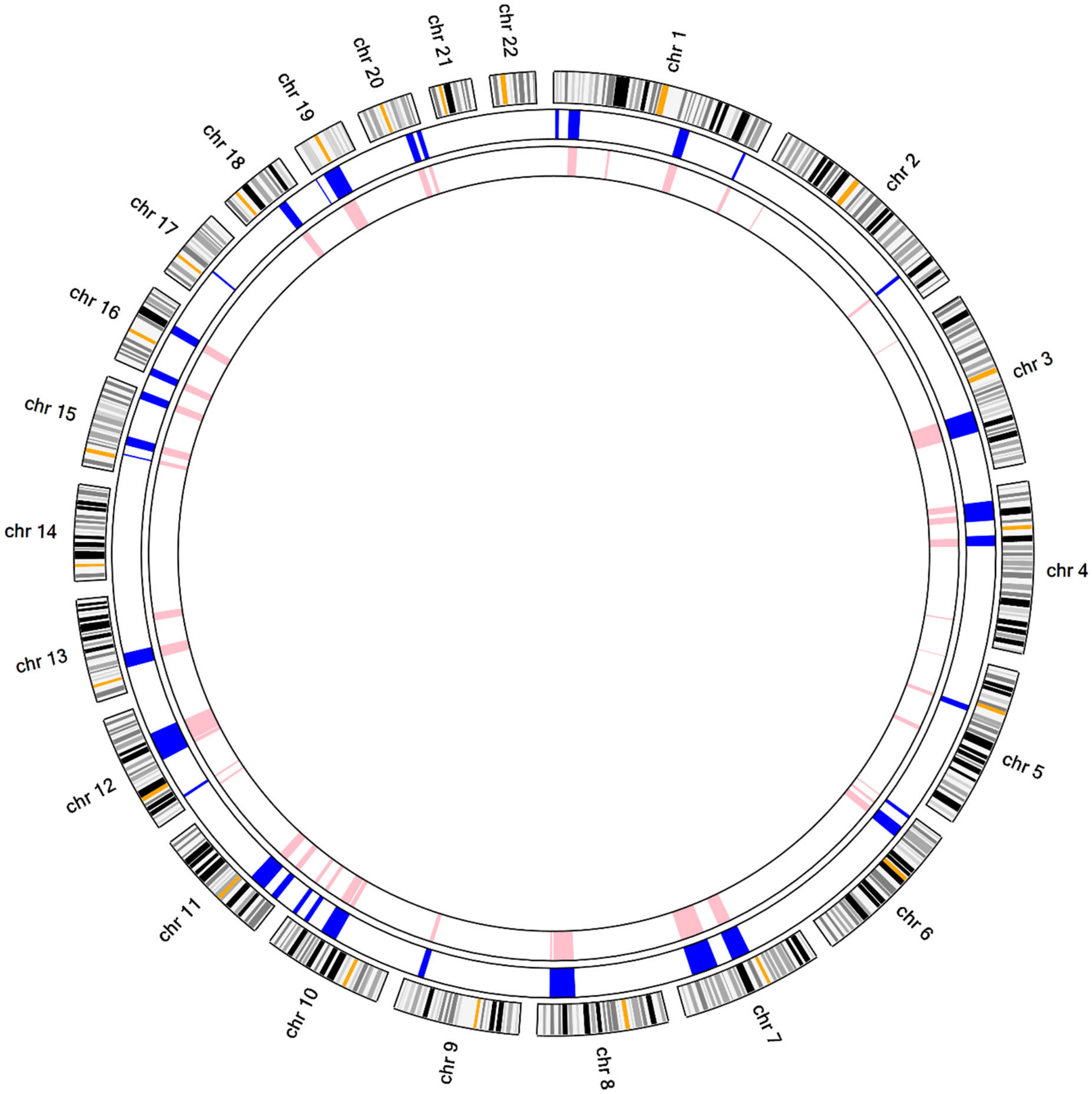

Supplement: Supplementary file 1 [file biology-14-00666-s001.zip › biology-3610721-supplementary.pdf]
